# Heterochiral DNA with Complementary Strands with α‐d and β‐d Configurations: Hydrogen‐Bonded and Silver‐Mediated Base Pairs with Impact of 7‐Deazapurines Replacing Purines

**DOI:** 10.1002/chem.202002765

**Published:** 2020-09-30

**Authors:** Yingying Chai, Xiurong Guo, Peter Leonard, Frank Seela

**Affiliations:** ^1^ Laboratory of Bioorganic Chemistry and Chemical Biology Center for Nanotechnology Heisenbergstrasse 11 48149 Münster Germany; ^2^ Department of Respiratory and Critical Care Medicine, Targeted Tracer Research and Development Laboratory West China Hospital Sichuan University 610041 Sichuan P. R. China; ^3^ Laboratorium für Organische und Bioorganische Chemie Institut für Chemie neuer Materialien Universität Osnabrück Barbarastrasse 7 49069 Osnabrück Germany

**Keywords:** chirality, DNA structures, nucleosides, oligonucleotides, silver

## Abstract

Heterochiral DNA with hydrogen‐bonded and silver‐mediated base pairs have been constructed using complementary strands with nucleosides with α‐d or β‐d configuration. Anomeric phosphoramidites were employed to assemble the oligonucleotides. According to the *T*_m_ values and thermodynamic data, the duplex stability of the heterochiral duplexes was similar to that of homochiral DNA, but mismatch discrimination was better in heterochiral DNA. Replacement of purines by 7‐deazapurines resulted in stable parallel duplexes, thereby confirming Watson–Crick‐type base pairing. When cytosine was facing cytosine, thymine or adenine residues, duplex DNA formed silver‐mediated base pairs in the presence of silver ions. Although the CD spectra of single strands with α‐d configuration display mirror‐like shapes to those with the β‐d configuration, the CD spectra of the hydrogen‐bonded duplexes and those with a limited number of silver pairs show a B‐type double helix almost indistinguishable from natural DNA. Nonmelting silver ion–DNA complexes with entirely different CD spectra were generated when the number of silver ions was equal to the number of base pairs.

## Graphical abstract

**Heterochiral DNA** with parallel strands has been constructed from complementary strands with nucleosides in the α‐d or β‐d configuration. Purines were replaced by 7‐deazapurines and silver‐mediated base pairs were formed when cytosine residues were facing cytosine, thymine, or adenine. The CD spectra of the modified helices show a B‐type structure almost indistinguishable from natural DNA.

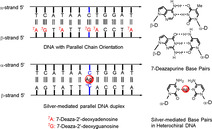

## Introduction

The DNA coding system originates from the selective recognition of complementary purines and pyrimidines. It goes back to Chargaff's observation that the sum of purines in DNA equals that of pyrimidines^[1]^ and to X‐ray investigations by Astbury,^[2]^ Franklin,^[3]^ and Wilkins.^[4]^ This resulted in the model of the right‐handed double helix proposed by Watson and Crick.^[5]^ dA pairs with dT and dG with dC. The double helix shows antiparallel strand orientation, and the strands are held together by hydrogen bonds and nucleobase stacking interactions. The sugar units of both strands display a β‐d conformation, and the strands have the same + helicity.^[6]^ Water molecules are highly important for the double helix‐structure, surrounding the DNA molecule and present in the grooves. Recognition occurs between two homochiral strands with identical sugar configurations.^[6]^

As the search for new orthogonal pairing systems continues in nucleic acid chemistry and biology, heterochiral nucleic acid strand recognition has been studied with the so‐called Spiegelmers, which consist of β‐l instead of β‐d nucleosides.^[7]^ Unfortunately, heterochiral hydrogen‐bonded nucleic acid strands, in which one strand displays the β‐d configuration and the other the β‐l configuration, do not form stable double helices.^[8]^

In contrast, oligonucleotides displaying the α‐d configuration in one strand and β‐d in the other hybridize and form stable duplexes.^[9]^ This pairing system was suggested in 1973 by Séquin and was based on Dreiding model studies.^[10]^ Later, it was shown that α‐d(CCTTCC) hybridizes with β‐d(GGAAGG) to form a duplex.^[9a]^ The parallel strand orientation was confirmed in the duplex α‐d(CATGCG)**⋅**β‐d(GTACGC).^[9d]^ Heterochiral duplexes of complementary α‐d and β‐d strands form a right‐handed double helix related to the B helix of DNA.[^9d^, ^9e^, ^9f^] Pioneering work in this field was reported by Imbach and co‐workers.[^9a^, ^9b^, ^9c^, ^9d^] Further studies on parallel DNA were undertaken by Hélène,^[9f]^ Morvan,[^9d^, ^11^] Germann,^[12]^ Debart,^[13]^ Jovin,^[14]^ Vasseur,^[15]^ and ourselves.^[16]^ From a synthetic point of view, the α/β heterochiral recognition system is demanding with respect to composition as eight building blocks, four α‐d nucleosides and four β‐d nucleosides, are necessary to construct a duplex instead of only four for canonical DNA.

**Figure 1:**
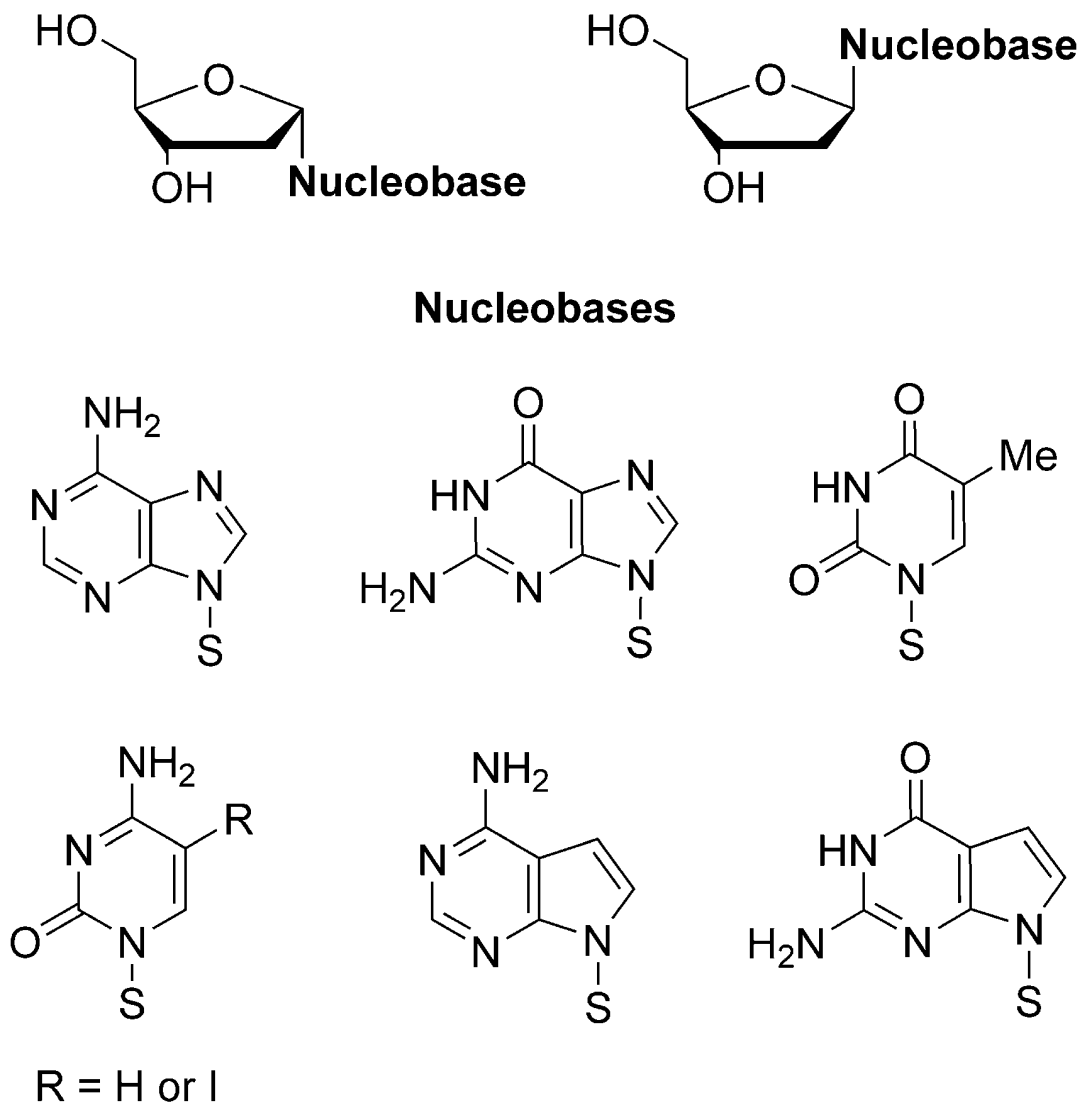
The α‐d and β‐d anomeric nucleosides used in this study.

Although α‐d nucleosides are not building blocks of canonical DNA, single α nucleosides have been detected in nucleic acids after irradiation under anoxic conditions.^[17]^ A few α‐d nucleosides are also naturally occurring.^[18]^ The required α‐d nucleosides are often side products in the convergent synthesis of nucleosides when glycosylation is not stereospecific. Special protocols have been developed to access α nucleosides,^[19]^ and all four building blocks have been synthesized.[^9a^, ^9b^] Figure 1 shows the structures of the nucleosides with canonical bases and 7‐deazapurine and pyrimidine modifications used in this study.

**Figure 2:**
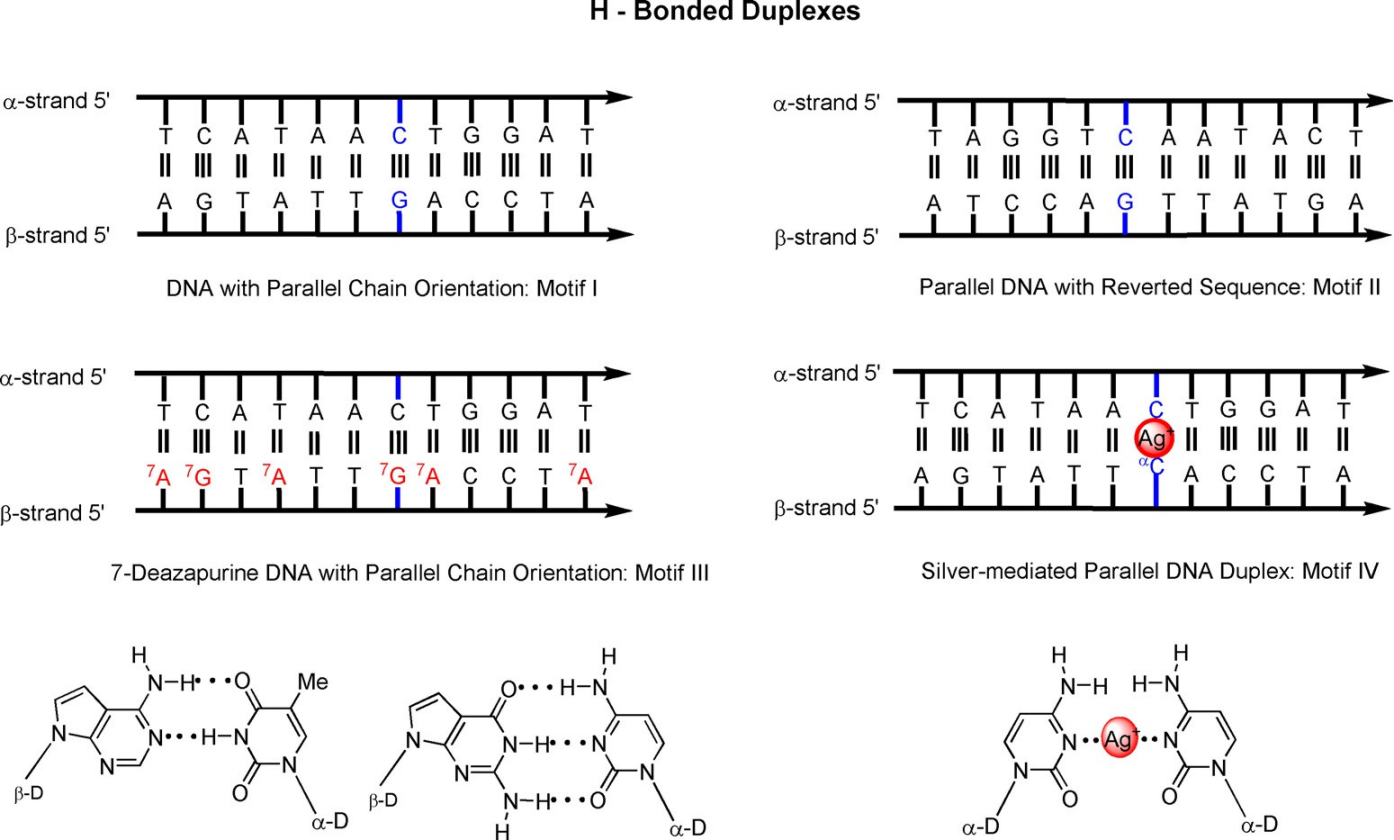
Schematic representation of the alignments of heterochiral duplexes with parallel and antiparallel chain orientation and DNA polarity changes of hydrogen‐bonded and silver‐mediated duplex DNA. T, A, G, C correspond to 2′‐deoxyribonucleosides and ^α^C corresponds to α‐2′‐deoxycytidine. ^7^A corresponds to 7‐deaza‐2′‐deoxyadenosine. ^7^G corresponds to 7‐deaza‐2′‐deoxyguanosine. The proposed hydrogen‐bonded and silver‐mediated base pairs are presented below.

Although a number of publications have appeared reporting on DNA with complementary heterochiral α/β strands or single or multiple heterochiral base pairs,[^9^, ^10^, ^11^, ^12^, ^13^, ^15^] knowledge of the function of modified nucleosides and base‐pair recognition in DNA with heterochiral strands is limited.^[20]^ Furthermore, no studies on mismatch formation and the stability of metal‐mediated base pairs have been reported previously. Therefore, we have carried out an investigation into heterochiral DNA displaying parallel strands. Hydrogen‐bonded and silver‐mediated base‐pair formation have been investigated with one strand displaying α‐d and the other the β‐d configuration. In more detail, DNA 12‐mer duplexes were constructed with and without mismatches and the duplex stability was determined according to changes in the *T*_m_ values. From these values, the thermodynamic data could be determined. Modified nucleosides, such as 7‐deazapurine and functionalized pyrimidine nucleosides, have been incorporated into the structures to replace canonical nucleosides. Homo‐ and heterochiral duplexes with reversed sequence polarity have been studied. In addition to 12‐mer duplexes, heterochiral 22‐mers were constructed, including DNA**⋅**RNA hybrids. Silver‐mediated base pairing of heterochiral DNA was investigated, and the stability of the metal‐mediated base pairs has been compared with hydrogen‐bonded base pairs in heterochiral DNA. To characterize the helical changes, CD spectra were recorded at defined temperatures or in variable‐temperature mode. The possible alignments with hydrogen‐bonded or silver‐mediated base pairs are displayed in Figure 2.

## Results and Discussion

### Synthesis and characterization of oligonucleotides for the construction of hetero‐ and homochiral DNA

The 12‐ and 22‐mer oligonucleotides used in this work were designed according to the standard duplex motif that has been used in our laboratory for many years to study base pairing with nucleoside analogues and to make data comparable within the various series. They were synthesized by employing solid‐phase phosphoramidite chemistry. α‐d‐ and β‐d‐phosphoramidites were either synthesized by us or were commercially available. The DNA synthesizer cycle was the same for α‐d oligonucleotides as for their β‐d counterparts. Even though the positions of the phosphoramidite residues in the α‐d nucleosides are in proximal positions to the nucleobase, no changes in the coupling times were required. After synthesis, the oligonucleotides were deprotected at 55 °C for 2 h in 28 % aq. NH_3_ and purified by reversed‐phase HPLC before and after detritylation. The purity of the oligonucleotides was proven by RP‐18 HPLC analysis (see Figures S2–S23 in the Supporting Information). The retention times for oligonucleotides with the same base composition but reversed sequences or containing α‐d instead of β‐d nucleosides were the same. Furthermore, the thermal hypochromicities measured between 25 and 75 °C were in the same range (18–20 %) for homo‐ and heterochiral duplexes with identical base composition. In accord with the change in the configuration, α‐d anomeric pairs of dA, dT, dG, and dC display different CD spectra.[^19c^, ^21^] They are not totally mirror‐like as they are pairs of diastereoisomers and not enantiomers. The sequences and mass spectral data of the oligonucleotides are displayed in Table 1.

**Table 1:** Synthesized oligonucleotides and their molecular masses determined by MALDI‐TOF MS.

Entry	Oligonucleotides	*M*_r,calcd_^[a]^ *M*_r,found_^[b]^	Entry	Oligonucleotides	*M*_r,calcd_^[a]^ *M*_r,found_^[b]^
ODN‐**1**	β‐5′‐d(TAGGTCAATACT)^[22]^	–	ODN‐**13**	β‐5′‐d(CAAACACCATTGTCACACTCCA)	6593.3 6590.9
ODN‐**2**	β‐5′‐d(AGTATTGACCTA)^[22]^	–	ODN‐**14**	α‐5′‐d(ACCTCACACTGTTACCACAAAC)	6593.3 6593.6
ODN‐**3**	β‐5′‐d(ATCCAGTTATGA)	3645.4 3645.9	ODN‐**15**	β‐5′‐r(UGGAGUGUGACAAUGGUGUUUG)	7116.3 7115.6
ODN‐**4**	β‐5′‐d(TCATAACTGGAT)	3645.4 3646.6	ODN‐**16**	β‐5′‐d(AGTATT^α^CACCTA)	3605.4 3603.3
ODN‐**5**	α‐5′‐d(TCATAACTGGAT)^[c]^	3644.4 3644.0	ODN‐**17**	β‐5′‐d(ACTATTCACCTA)	3565.4 3564.7
ODN‐**6**	α‐5′‐d(TAGGTCAATACT)^[c]^	3644.4 3644.0	ODN‐**18**	β‐5′‐d(A^α^CTATT^α^CACCTA)	3565.4 3564.9
ODN‐**7**	β‐5′‐d(AGTATTCACCTA)	3605.4 3603.9	ODN‐**19**	β‐5′‐d(AGTATT1ACCTA)^[23]^	3731.3 3730.5
ODN‐**8**	β‐5′‐d(AGTATTAACCTA)	3629.4 3629.2	ODN‐**20**	β‐5′‐d(AGTATT2ACCTA)^[23]^	3731.3 3730.2
ODN‐**9**	β‐5′‐d(AGTATTTACCTA)	3620.4 3619.3	ODN‐**21**	β‐5′‐d(ATCCACTTATGA)	3605.4 3604.2
ODN‐**10**	β‐5′‐d(^7^A^7^GT^7^ATT^7^G^7^ACCT^7^A)	3639.5 3638.5	ODN‐**22**	β‐5′‐d(ATCCA^α^CTTATGA)	3605.4 3604.0
ODN‐**11**	β‐5′‐d(T^7^A^7^G^7^GTC^7^A^7^AT^7^ACT)	3639.5 3639.1	ODN‐**23**	β‐5′‐d(TAGGTAAATACT)^[22]^	–
ODN‐**12**	β‐5′‐d(TGGAGTGTGACAATGGTGTTTG)	6877.5 6875.9	ODN‐**24**	β‐5′‐d(AGTATTTACCTA)^[22]^	–

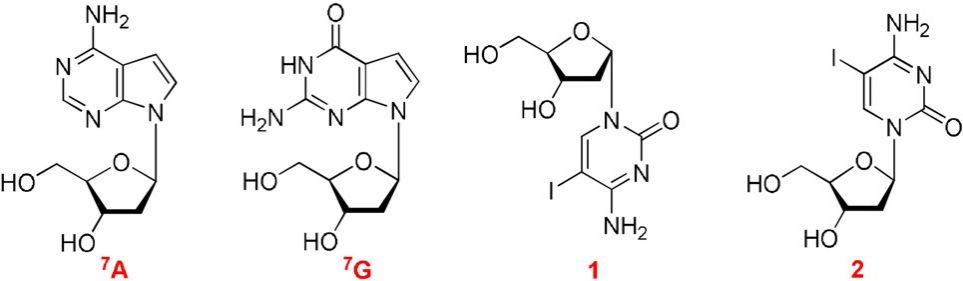					

[a] Calculated on the basis of the molecular mass of [*M*+H]^+^. [b] Determined by MALDI‐TOF MS as [*M*+H]^+^ in the linear positive mode. [c] Calculated on the basis of the molecular mass [*M*]^+^ and measured by MALDI‐TOF MS in the linear negative mode. ^7^A corresponds to 7‐deaza‐2′‐deoxyadenosine, ^7^G corresponds to 7‐deaza‐2′‐deoxyguanosine, 1 corresponds to 5‐iodo‐2′‐deoxycytidine, and 2 corresponds to α‐5‐iodo‐2′‐deoxycytidine.^[23]^

### Hetero‐ and homochiral oligonucleotide duplexes with hydrogen‐bonded base pairs

In the first part of the investigation, the thermal stabilities of a series of duplexes with homo‐ and heterochiral strand alignment were studied. To this end, 12‐mer duplexes were constructed by the hybridization of two complementary β strands or complementary strands with one strand with the α‐d configuration and the other with β‐d. More specifically, the 12‐mer homochiral duplex β‐5′‐d(TAGGTCAATACT)**⋅**β‐5′‐d(AGTATTGACCTA) ((ODN‐**1**)**⋅**(ODN‐**2**)), showing the antiparallel chain orientation, was used as a reference compound. In the heterochiral duplex with parallel chain orientation in one of the two strands, all β‐d nucleosides were replaced by their α‐d anomers, resulting in the heterochiral duplex α‐5′‐d(TCATAACTGGAT)**⋅**β‐5′‐d(AGTATTGACCTA) ((ODN‐**5**)**⋅**(ODN‐**2**)).

According to the parallel chain alignment of the heterochiral duplex, the α strand displays a sequence that is reversed from the 5′ to the 3′ terminus with respect to the β strand (ODN‐**1**) in the homochiral duplex. In addition, homo‐ and heterochiral duplexes were constructed in which the sequences were reversed in both strands (Figure 2). The latter change should reveal whether hetero‐ and homochiral duplexes with reversed sequences exhibit similar behavior with regard to base pairing and duplex stability. The 22‐mer duplexes were designed in a similar way but with the aim to be complementary to a particular microRNA (miRNA‐122).

Hetero‐ and homochiral duplexes were modified in the following ways: 1) By reversion of the sequences of both strands with otherwise identical base composition, according to Figure 2, 2) by incorporation of mismatches into the hetero‐ and homochiral duplexes in which the cytosine base is positioned opposite the four canonical bases, and 3) by replacement of purine bases by 7‐deazapurines or dC by α‐dC, 5‐iodo‐dC, or α‐5‐iodo‐dC.

Then, the thermal stabilities of the duplexes were determined by *T*_m_ measurements. The thermodynamic data (Δ*G*, Δ*H*, and Δ*S*) were calculated from the *T*_m_ values. CD spectra were measured and temperature‐dependent profiles were collected to obtain information on the helical changes in the single strands and duplexes.

**Figure 3:**
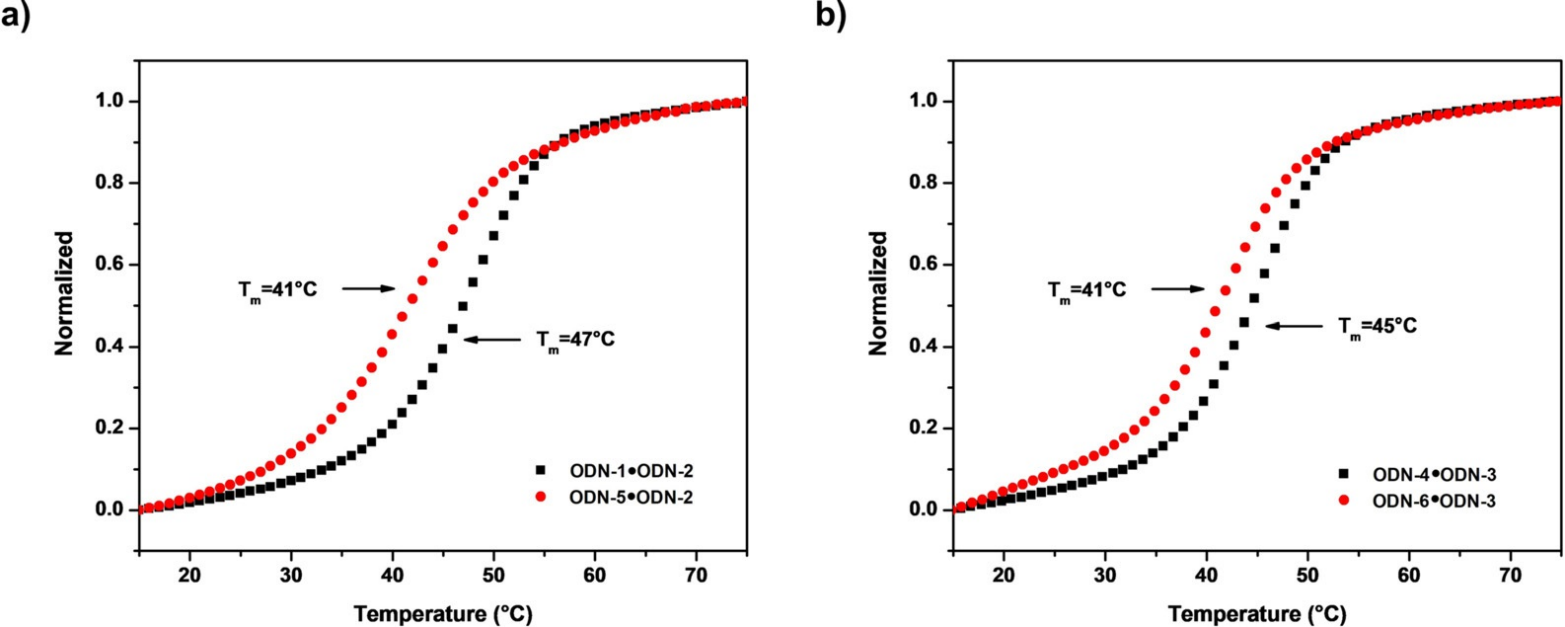
Thermal denaturation experiments performed with 5 μm+5 μm single‐strand concentrations in buffer solution (100 mm NaOAc, 10 mm Mg(OAc)_2_, pH 7.4) detected at 260 nm: a) ODN‐**1⋅**ODN‐**2** (black) and ODN‐**5⋅**ODN‐**2** (red); b) ODN‐**6⋅**ODN‐**3** (red) and ODN‐**4⋅**ODN‐**3** (black).

Figure 3 a displays the typical melting profiles of the 12‐mer duplexes ODN‐**1⋅**ODN‐**2** and ODN‐**5⋅**ODN‐**2**. The corresponding data, including those of related duplexes, are presented in Table 2. It is evident that heterochiral duplexes with parallel chain orientation are slightly less stable (lower *T*_m_ values) than homochiral duplexes with antiparallel chains. This phenomenon is base‐dependent and corresponds to less negative Δ*G* values resulting from an unfavorable enthalpic term for α/β duplexes compared with β/β strands, whereas the entropy is more favorable.^[11]^ Figure 3 b shows the melting profiles of the reversed duplexes. The heterochiral duplexes display identical *T*_m_ values with respect to the nonreversed (“original”) duplexes, and almost no influence on the enthalpy and entropy data is observed, although they are not identical. For the homochiral duplexes, a slightly lower *T*_m_ value associated with a gain in enthalpy that is overcompensated by a loss in entropy for the reversed and non‐“original” duplexes is observed.

**Table 2:** *T*_m_ values and thermodynamic data for parallel heterochiral (one strand α‐d/one strand β‐d) and antiparallel homochiral (both strands β‐d) oligonucleotide duplexes.^[a]^

Heterochiral duplex, parallel strands	*T*_m_ [°C]	Δ*H*_310_ [kcal mol^−1^]	Δ*S*_310_ [cal K^−1^ mol^−1^]	Δ*G*_310_ [kcal mol^−1^]
α‐5′‐d(TAGGTCAATACT) (ODN‐**6**) β‐5′‐d(ATCCAGTTATGA) (ODN‐**3**)	41	−63.3	−174.6	−9.1
α‐5′‐d(TCATAACTGGAT) (ODN‐**5**) β‐5′‐d(AGTATTGACCTA) (ODN‐**2**)	41	−65.0	−180.0	−9.1

Homochiral duplex, antiparallel strands	*T*_m_ [°C]	Δ*H*_310_ [kcal mol^−1^]	Δ*S*_310_ [cal K^−1^ mol^−1^]	Δ*G*_310_ [kcal mol^−1^]
β‐5′‐d(TAGGTCAATACT) (ODN‐**1**) β‐3′‐d(ATCCAGTTATGA) (ODN‐**2**)	47	−81.8	−228.3	−11.0
β‐5′‐d(TCATAACTGGAT) (ODN‐**4**) β‐3′‐d(AGTATTGACCTA) (ODN‐**3**)	45	−85.8	−242.9	−10.5

[a] Measured at 260 nm with 5 μm+5 μm single‐strand concentrations at a cooling rate of 1.0 °C min^−1^ in 100 mm NaOAc and 10 mm Mg(OAc)_2_ (pH 7.4). *T*_m_ values were calculated from the cooling curves using the Meltwin 3.0 program.^[25]^

Heterochiral α/β duplexes can be more stable than canonical duplexes when the α strand contains more pyrimidines than purines.^[11]^ An increasing number of purines reduces duplex stability. In our 12‐mer duplexes, the number of purines and pyrimidines is balanced (six purines and six pyrimidines). The stability of these heterochiral duplexes is only slightly lower than that of their homochiral counterparts. Apparently, the base composition, in combination with the anomeric centers of the purine and pyrimidine nucleosides within the two strands, plays a major role in this behavior.

**Figure 4:**
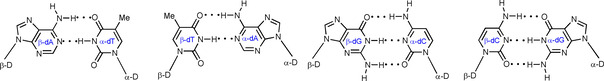
The four base‐pair combinations of complementary α/β‐d nucleosides in parallel α/β DNA duplexes.

The composition will affect the backbone geometry. In more detail, the torsion angles around the glycosylic bonds of the α‐d and β‐d anomeric residues are different for purine and pyrimidine nucleosides, but also for pairs of anomeric nucleosides.^[24]^ This can strengthen or weaken a base pair. Thus, conformational restriction around the glycosylic bond of α and β anomers might be the main reason for the base‐dependent changes caused by purine and pyrimidine nucleosides. We anticipate that four different base pairs are formed in heterochiral DNA, and these are shown in Figure 4. By contrast, in homochiral β/β DNA, only two base pairs are possible, namely β‐dA**⋅**β‐dT and β‐dG**⋅**β‐dC.

Nevertheless, the specificity of base recognition is the same in hetero‐ and homochiral DNA and follows the Watson–Crick principle. This contrasts with other parallel DNA formed by iG_d_ **⋅**dC, iC_d_ **⋅**dG (iG_d_=2′‐deoxyisoguanosine, iC_d_=2′‐deoxyisocytidine), and reversed Watson–Crick dA**⋅**dT pairs. In this case, the parallel alignment of the strands in the double helix is evoked by the change in the recognition sites of the nucleobases.[^14^, ^16^]

**Figure 5:**
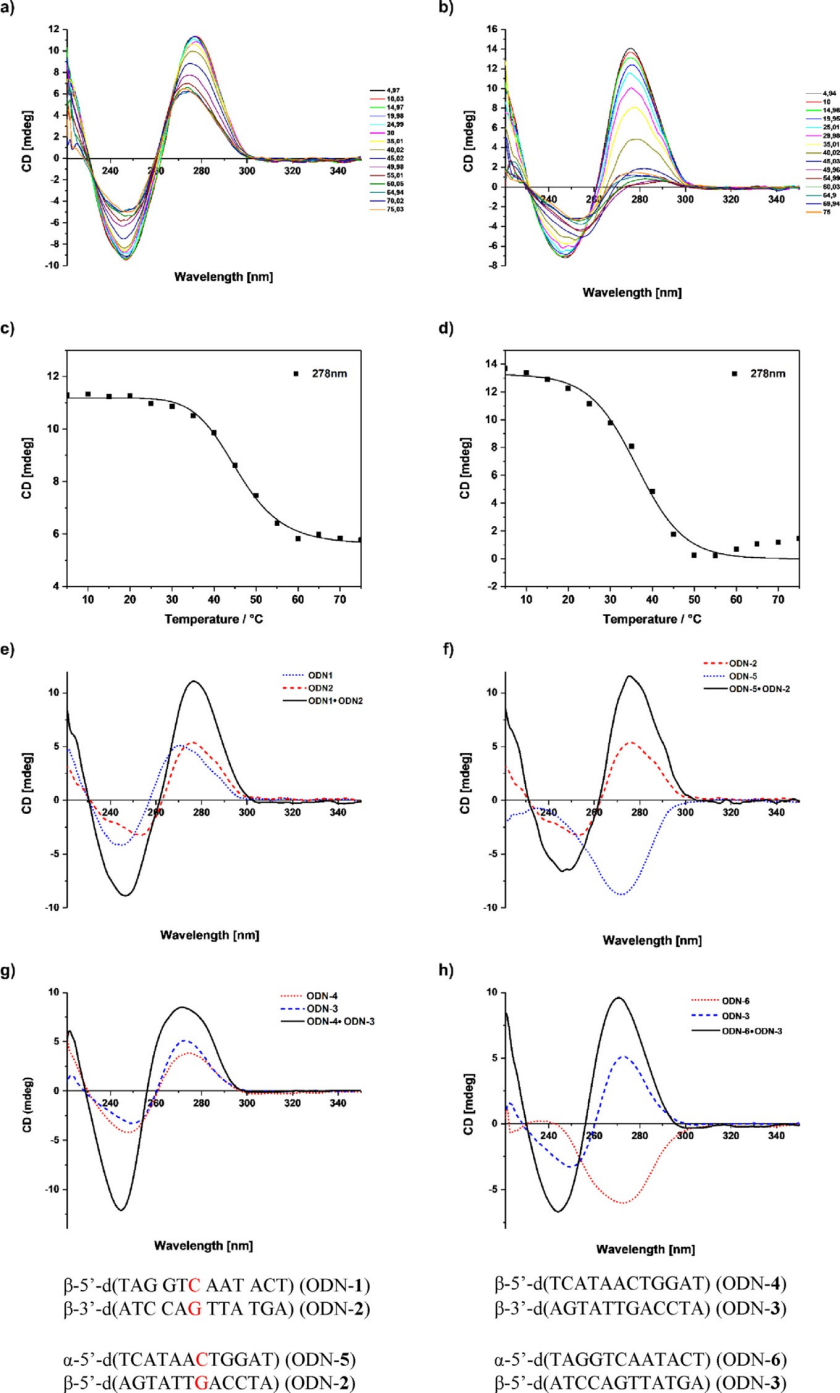
Temperature‐dependent CD spectra of the oligodeoxyribonucleotides a) ODN‐**1⋅**ODN‐**2** and b) ODN‐**5⋅**ODN‐**2**. CD melting curves obtained from the temperature‐dependent CD spectra of c) ODN‐**1⋅**ODN‐**2** (β/β) and d) ODN‐**5⋅**ODN‐**2** (α/β). CD spectra of e) ODN‐**1**, ODN‐**2**, and ODN‐**1⋅**ODN**‐2**, f) ODN‐**2**, ODN‐**5**, and ODN‐**5⋅**ODN**‐2**, g) ODN‐**3**, ODN‐**4**, and ODN‐**3⋅**ODN**‐4**, and h) ODN‐**3**, ODN‐**6**, and ODN‐**6⋅**ODN**‐3**. All measurements were performed in 100 mm NaOAc/10 mm Mg(OAc)_2_ buffer (pH 7.4). The cell path length of the cuvette used to record the CD spectra was 1.0 cm.

CD spectra were recorded to determine the structural differences in the homo‐ and heterochiral duplexes. Figure 5 a,b displays the evolution of the CD spectra during melting, and Figure 5 c,d shows the CD melting curves. The spectra at low temperature indicate that both duplexes form a B‐type DNA structure. Clearly, the β‐d strand dictates the double‐helix structure. This is not unique for this particular duplex but is a general finding for all duplexes formed by a combination of α‐d and β‐d strands.^[11]^ The CD maximum of the positive lobe of the homochiral duplex ODN‐**1⋅**ODN‐**2** is located at 278 nm and that of the negative lobe at 248 nm. As the temperature is increased, the maximum of the positive lobe is shifted hypsochromically (ca. 3 nm), whereas the negative lobe shows no shift. Furthermore, with increasing temperature, the amplitude of the lobe at 278 nm becomes smaller as more single strands are formed. Two isosbestic points are observed at 230 and 265 nm.

For the heterochiral α/β duplex ODN‐**5⋅**ODN‐**2** the situation is different. The change in the positive lobe of the CD spectrum upon heating is much more marked as it almost completely disappears upon melting. The maximum of the negative lobe is bathochromically shifted and only one isosbestic point is observed. These changes are a result of the differences in the CD spectra of the oligonucleotide single strands (α‐d vs. β‐d), which show mirror‐like spectra. This contrasts with the homochiral duplex ODN‐**1⋅**ODN‐**2**, as the single strands of ODN‐**1** and ODN‐**2** exhibit a similar helicity (Figure 5 e,f). Further heating leads to a continuous increase in absorbance due to a decrease of stacking interactions.

As discussed above, we have constructed duplexes with reversed sequences in both strands, measured the *T*_m_ values of the corresponding homo‐ and heterochiral duplexes, and compared them to each other. The CD spectrum of the reversed α/β duplex ODN‐**6⋅**ODN‐**3** (Figure 5 h) shows only marginal differences with the spectrum of the “original” duplex (Figure 5 f). An increased amplitude height of the positive lobe is combined with a small (ca. 3 nm) hypsochromic shift, whereas the negative lobe is unchanged. The CD spectrum of the reversed β/β duplex ODN‐**4⋅**ODN‐**3** shows more pronounced changes (Figure 5 g). The amplitude of the positive lobe is reduced, the width is broadened, and the maximum is hypsochromically shifted (ca. 3 nm). The negative lobe is narrower and the amplitude height is increased. According to the CD spectra and *T*_m_ values, the structures of the reversed and “original” duplexes are similar in the case of the α/β duplexes (Figure 5 h), whereas there are some differences for the β/β duplexes. Probably, the changes in the oligonucleotide sequence are better tolerated in parallel heterochiral duplexes than in antiparallel homochiral duplexes.

In the next experiments, mismatch discrimination of heterochiral DNA was studied with respect to homochiral DNA. A better mismatch discrimination of heterochiral duplexes would represent an interesting feature with respect to the detection of genetic errors occurring during replication or caused by irradiation or other environmental events. In general, mismatches are less stable than canonical bidentate or tridentate base pairs. Nevertheless, the mismatches can form hydrogen bonds and their stability varies significantly among the various base combinations. A significant difference in duplex stability between matches and mismatches is required to distinguish false positives and false negatives to detect genomic errors in DNA. In diagnostic probes a high *T*_m_ difference in base‐pair matches and mismatches is required to obtain data with confidence. To this end, the four canonical bases were positioned opposite dC in homo‐ and heterochiral DNA. The formation of the cytosine–cytosine mismatch was of particular interest for silver‐mediated base‐pair formation, discussed in the next section. According to Table 3 and the UV melting profiles presented in Figure S24 in the Supporting Information, there are larger differences in *T*_m_ values between mismatches in heterochiral (α/β) DNA than in homochiral (β/β) DNA. The temperature decrease of one mismatch in heterochiral duplexes is around 30 °C, but only around 20 °C in their homochiral counterparts. Even considering the fact that the *T*_m_ values of heterochiral DNA are lower than those of homochiral DNA, mismatch discrimination is significantly better in the heterochiral α/β system than in canonical β/β duplexes. This might be due to stronger conformational changes or the loss of stacking of nearest‐neighbor nucleosides in the heterochiral system.

**Table 3:** *T*_m_ values of parallel heterochiral (one strand α‐d/one strand β‐d) and antiparallel homochiral (both strands β‐d) oligonucleotide duplexes.^[a]^

Heterochiral duplex, parallel strands	*T*_m_ [°C]	Homochiral duplex, antiparallel strands	*T*_m_ [°C]
α‐5′‐d(TCATAACTGGAT) (ODN‐**5**) β‐5′‐d(AGTATTGACCTA) (ODN‐**2**)	41	β‐5′‐d(TAGGTCAATACT) (ODN‐**1**) β‐3′‐d(ATCCAGTTATGA) (ODN‐**2**)	47
α‐5′‐d(TCATAACTGGAT) (ODN‐**5**) β‐5′‐d(AGTATTTACCTA) (ODN‐**9**)	<15^[b]^	β‐5′‐d(TAGGTCAATACT) (ODN‐**1**) β‐3′‐d(ATCCATTTA GA) (ODN‐**9**)	26
α‐5′‐d(TCATAACTGGAT) (ODN‐**5**) β‐5′‐d(AGTATTAACCTA) (ODN‐**8**)	<15^[b]^	β‐5′‐d(TAGGTCAATACT) (ODN‐**1**) β‐3′‐d(ATCCAATTATGA) (ODN‐**8**)	25
α‐5′‐d(TCATAACTGGAT) (ODN‐**5**) β‐5′‐d(AGTATTCACCTA) (ODN‐**7**)	<15^[b]^	β‐5′‐d(TAGGTCAATACT) (ODN‐**1**) β‐3′‐d(ATCCACTTATGA) (ODN‐**7**)	27

[a] Measured at 260 nm with 5 μm+5 μm single‐strand concentrations at a cooling rate of 1.0 °C min^−1^ in 100 mm NaOAc and 10 mm Mg(OAc)_2_ (pH 7.4). [b] Exact *T*_m_ values for duplexes with heterochiral mismatches could not be detected as the duplexes were too short to show complete melting profiles in the range 15–50 °C (see Figure S24 in the Supporting Information). *T*_m_ values were calculated from the cooling curves using the Meltwin 3.0 program.^[25]^

DNA incorporating 7‐deazapurines is commonly used for structural and functional investigation of nucleic acids in chemistry and biology. The stereoselective synthesis of 7‐deazapurine nucleosides as well as their incorporation into oligonucleotides by chemical solid‐phase synthesis and with polymerases was discovered in our laboratory.^[26]^ Due to the absence of N‐7, these nucleosides cannot form Hoogsteen base pairs and do not bind ligands, including metal ions, in the major groove of DNA. Many modifications on nucleoside 7‐deazapurine triphosphates have now been performed to make use of this phenomenon.^[27]^ Moreover, because modifications at N‐7 of these nucleosides are well accommodated in DNA, 7‐deazapurine nucleosides have found widespread application in chemistry and chemical biology,^[27]^ for example, 7‐deazapurine nucleosides have been used in the Sanger sequencing protocol using fluorescent dyes or in sequencing by synthesis.^[27]^

**Figure 6:**
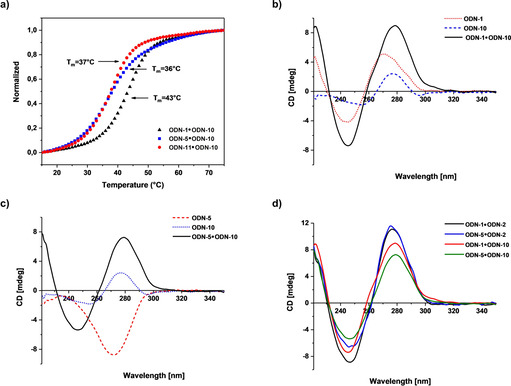
a) Thermal melting curves of ODN‐**1⋅**ODN‐**10** (β/β, black), ODN‐**5⋅**ODN‐**10** (α/β, blue), and ODN‐**10⋅**ODN‐**11** (β/β, red). CD spectra of b) ODN‐**1**, ODN‐**10**, and ODN‐**1⋅**ODN**‐10**, c) ODN‐**5**, ODN‐**10**, and ODN‐**5⋅**ODN**‐10**, and d) ODN‐**1⋅**ODN‐**2**, ODN‐**5⋅**ODN‐**2**, ODN‐**1⋅**ODN‐**10**, and ODN‐**5⋅**ODN‐**10**. All measurements were performed with a 5 μm+5 μm single‐strand concentrations in 100 mm NaOAc and 10 mm Mg(OAc)_2_ (pH 7.4). The cell path length of the cuvette used to record the CD spectra was 1.0 cm.

Thus, a large body of information exists on the functionalization of 7‐deazapurine nucleosides in canonical DNA. However, nothing is known on their impact on heterochiral DNA formed by complementary strands with α‐d and β‐d nucleoside configurations. To this end, all purine nucleosides of the β strand of heterochiral DNA were replaced by 7‐deazapurine residues, while the α strand was left untouched. Accordingly, duplex ODN‐**5⋅**ODN‐**10** contains four c^7^A_d_ **⋅**dT and two c^7^G_d_ **⋅**dC base pairs as well as two dG**⋅**dC and four dA**⋅**dT pairs. Figure 6 a shows the melting profiles of the base‐modified duplexes. The *T*_m_ values and thermodynamic data are summarized in Table 4.

**Table 4:** *T*_m_ values and thermodynamic data of parallel heterochiral (one strand α‐d/one strand β‐d) and antiparallel homochiral (both strands β‐d) oligonucleotide duplexes.^[a]^

Heterochiral duplex, parallel strands	*T*_m_ [°C]	Δ*H*_310_ [kcal mol^−1^]	Δ*S*_310_ [cal K^−1^ mol^−1^]	Δ*G*_310_ [kcal mol^−1^]
α‐5′‐d(TCATAACTGGAT) (ODN‐**5**) β‐5′‐d(AGTATTGACCTA) (ODN‐**2**)	41	−65.0	−180.0	−9.1
α‐5′‐d(TCATAACTGGAT) (ODN‐**5**) β‐5′‐d(^7^A^7^GT^7^ATT^7^G^7^ACCT^7^A) (ODN‐**10**)	36	−60.5	−169.0	−8.1

Homochiral duplex, antiparallel strands	*T*_m_ [°C]	Δ*H*_310_ [kcal mol^−1^]	Δ*S*_310_ [cal K^−1^ mol^−1^]	Δ*G*_310_ [kcal mol^−1^]
β‐5′‐d(TAGGTCAATACT) (ODN‐**1**) β‐3′‐d(ATCCAGTTATGA) (ODN‐**2**)	47	−81.8	−228.3	−11.0
β‐5′‐d(TAGGTCAATACT) (ODN‐**1**) β‐3′‐d(^7^ATCC^7^A^7^GTT^7^AT^7^G^7^A) (ODN‐**10**)	43	−84.4	−230.4	−9.9
β‐5′‐d(T^7^A^7^G^7^GTC^7^A^7^AT^7^ACT) (ODN‐**11**) β‐3′‐d(^7^ATCC^7^A^7^GTT^7^AT^7^G^7^A) (ODN‐**10**)	37	−73.3	−209.3	−8.3

[a] Measured at 260 nm with 5 μm+5 μm single‐strand concentrations at a cooling rate of 1.0 °C min^−1^ in 100 mm NaOAc and 10 mm Mg(OAc)_2_ (pH 7.4). *T*_m_ values were calculated from the cooling curves using the Meltwin 3.0 program.^[25]^ ^7^A corresponds to 7‐deaza‐2′‐deoxyadenosine and ^7^G corresponds to 7‐deaza‐2′‐deoxyguanosine (for structures, see Table 1).

The data in Table 4 clearly show that the 7‐deazapurine nucleosides in homochiral DNA destabilize the double helix by about 10 °C compared with the duplex formed entirely by purine bases. The *T*_m_ is higher when only one strand contains purines and the other 7‐deazapurines. This destabilization together with the absence of N‐7 is an important factor for their application in nucleic acid chemistry and biology. The heterochiral duplex ODN‐**5⋅**ODN‐**10**, in which all the purines of one strand are replaced by 7‐deazapurines, shows a similar difference in *T*_m_ as its homochiral counterpart. A comparison of the thermodynamic data for duplexes containing 7‐deazapurines and purines also reveals differences. In the α/β duplex, an enhanced entropy term is compensated by a decreased enthalpy, whereas in the β/β duplex a strong gain in enthalpy is compensated by unfavorable entropy. Furthermore, the distribution of 7‐deazapurine**⋅**pyrimidine base pairs in DNA confirms that base pairing in heterochiral duplexes follows the Watson–Crick mode and Hoogsteen pairing can be excluded. The experiments performed with 7‐deazapurine nucleosides indicate that this type of base modification in homochiral DNA can be adapted to heterochiral DNA. Furthermore, they demonstrate the similarities of the parallel and antiparallel duplex structures, with both showing a B‐type double helix. To the best of our knowledge, this is also the first example of an α strand hybridizing to a base‐modified β strand and forming a stable duplex.

Next, the CD spectra of 12‐mer duplexes containing 7‐deazapurines were recorded at defined temperatures (Figure 6 b–d). First, the spectra of the single strands were recorded. The modified β strand containing 7‐deazapurines (ODN‐**10**) shows a shows a bathochromic shift (ca. 10 nm) along with a diminished amplitude of the positive lobe compared with the corresponding purine strand (ODN‐**1**; Figure 6 b). The negative lobe is bathochromically shifted (ca. 10 nm) and its amplitude is also reduced. The situation changes completely when the duplexes are formed. The CD spectra of homo‐ and heterochiral duplexes containing 7‐deazapurines instead of purines are almost identical and display structural features of the B‐DNA form (Figure 6 d). Thus, in the case of heterochiral α/β duplexes containing 7‐deazapurines, the duplex structure is controlled by the β strand (Figure 6 c).

**Figure 7:**
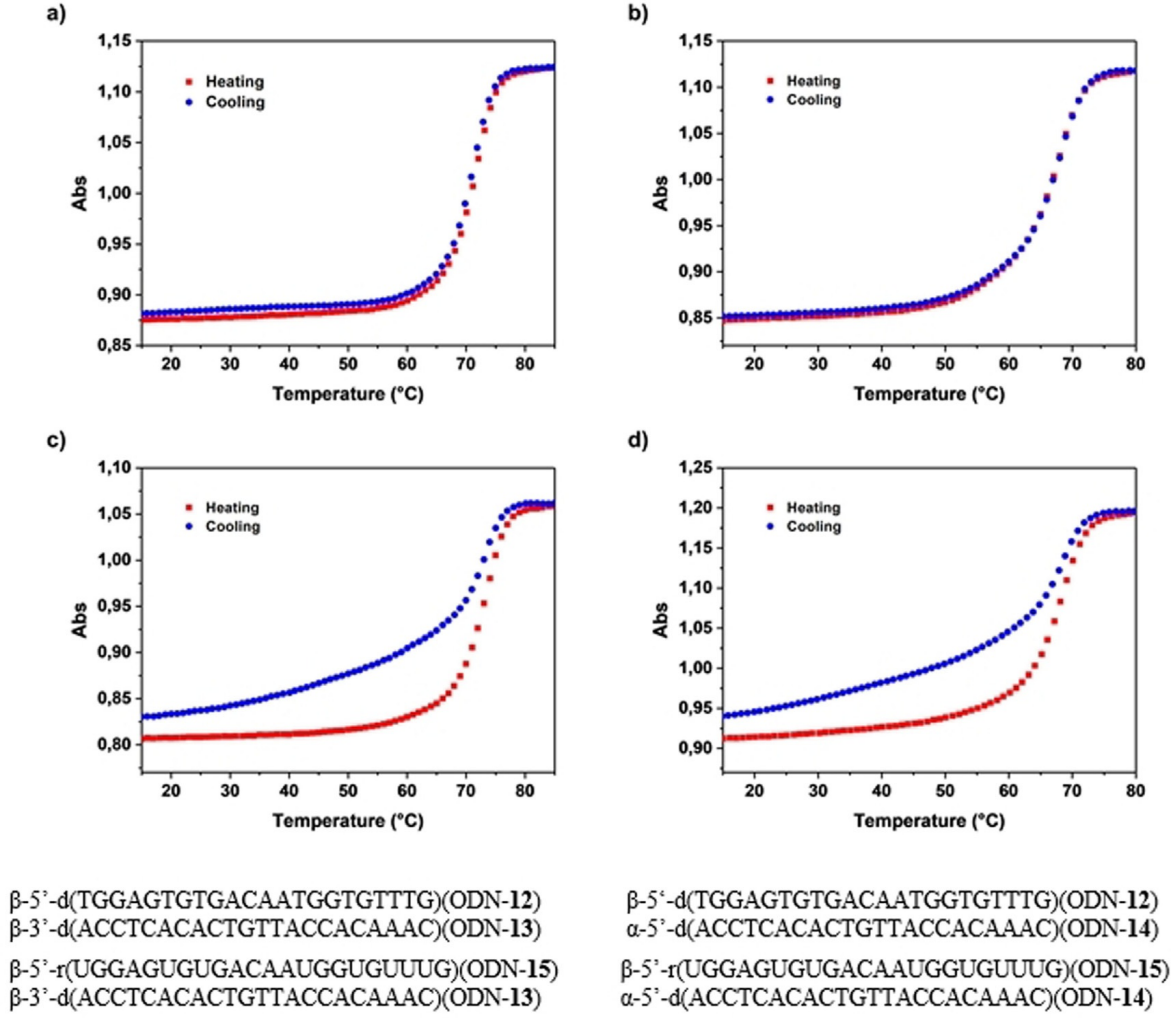
Thermal denaturation experiments of duplexes a) ODN‐**12⋅**ODN‐**13**, b) ODN‐**12⋅**ODN‐**14**, c) ODN‐**15⋅**ODN‐**13**, and d) ODN‐**14⋅**ODN‐**15**. All measurements were performed with 5 μm+5 μm single‐strand concentrations in 100 mm NaOAc and 10 mm Mg(OAc)_2_ (pH 7.4).

It has been reported that α‐d oligonucleotides are able to hybridize to β‐d RNA strands to form stable duplexes. The stability depends on the base composition.[^11^, ^28^] From this, applications relating to the intracellular inhibition of microRNAs can be expected. For this, the 22‐mer 2′‐deoxyribooligonucleotide ODN‐**14** consisting of α‐d nucleosides was synthesized. Hybridization with the target microRNA‐122 (ODN‐**15**) gave a stable duplex (*T*_m_=68 °C; Table 5 and Table S1 in the Supporting Information), which is almost as stable as the homochiral RNA**⋅**DNA hybrid ODN‐**15⋅**ODN‐**13** (*T*_m_=73 °C). Apparently, the microRNA binds strongly to the α strand to form an RNA**⋅**DNA hybrid with parallel chain orientation. Consequently, α oligonucleotides have the potential to inhibit the activity of microRNAs in cellular processes in which cleavage of RNA is not required. For comparison, the corresponding DNA**⋅**DNA duplexes were measured. A significant difference exists between the DNA**⋅**DNA and DNA**⋅**RNA duplexes. According to the melting curves shown in Figure 7, the DNA melting profiles of both RNA**⋅**DNA hybrids show hysteresis of the curves during the cooling process, whereas the DNA**⋅**DNA duplexes do not. This is typical of DNA**⋅**RNA hybrids in which the RNA strand can form secondary structures and the alignment of the two strands requires the reorganization of the RNA strand. Both the hetero‐ and homochiral duplexes show this behavior as they contain the same RNA strand.

**Table 5:** *T*_m_ values of parallel heterochiral (one strand α‐d/one strand β‐d) and antiparallel homochiral (β/β‐d) oligonucleotide duplexes.^[a]^

Duplexes	*T*_m_ [°C]	Duplexes	*T*_m_ [°C]
β‐5′‐d(TGGAGTGTGACAATGGTGTTTG) (ODN‐**12**) α‐5′‐d(ACCTCACACTGTTACCACAAAC) (ODN‐**14**)	68	β‐5′‐d(TGGAGTGTGACAATGGTGTTTG) (ODN‐**12**) β‐3′‐d(ACCTCACACTGTTACCACAAAC) (ODN‐**13**)	71
β‐5′‐r(UGGAGUGUGACAAUGGUGUUUG) (ODN‐**15**) α‐5′‐d(ACCTCACACTGTTACCACAAAC) (ODN‐**14**)	68	β‐5′‐r(UGGAGUGUGACAAUGGUGUUUG) (ODN‐**15**) β‐3′‐d(ACCTCACACTGTTACCACAAAC) (ODN‐**13**)	73

[a] Measured at 260 nm with 5 μm+5 μm single‐strand concentrations at a heating rate of 1.0 °C min^−1^ in 0.1 m NaCl, 10 mm MgCl_2_, and 10 mm Na cacodylate. *T*_m_ values were calculated from the heating curves using the Meltwin 3.0 program.^[25]^

### Hetero‐ and homochiral duplexes with silver‐mediated base‐pair mismatches at low silver ion concentration

In 2004, Ono and co‐workers discovered that the dC**⋅**dC mismatch selectively binds silver ions to form stable silver‐mediated base pairs.^[29]^ Later, they reported a single‐crystal X‐ray analysis showing that the silver ions bridge the two N‐3 atoms of the dC**⋅**dC mismatch.^[30]^ Extremely stable pyrrolo**⋅**dC and imidazolo**⋅**dC pairs were constructed in our laboratory.^[31]^

The formation of silver‐mediated c^7^G_d_ **⋅**dC and c^7^A_d_ **⋅**dT pairs has been reported^[32]^ and also a crystallized DNA silver wire containing silver‐mediated canonical base pairs.^[33]^ Recently, we showed that the anomeric heterochiral α‐dC**⋅**β‐dC pair is more stable than the homochiral β‐dC**⋅**β‐dC and α‐dC**⋅**α‐dC pairs.[^23^, ^34^] The number of bound silver ions per base pair was determined by spectrophotometric titration: One or two silver ions were found to be present in the metal‐mediated base pairs. Furthermore, it was observed that not only dC**⋅**dC mismatches form silver‐mediated base pairs, because stable duplexes were also generated after the addition of silver ions to mismatches of dC with dT,[^34^, ^35^] dA,^[34]^ or 7‐deaza‐dA.^[36]^ It was found that the increase in *T*_m_ for 12‐mer duplexes caused by silver‐mediated base pairs of dC with dA or dT was in the same range as that of the dC–Ag^+^–dC pair. Also, successful DNA polymerase‐catalyzed primer extension mediated by silver ions by the formation of dC–Ag^+^ pairs with dA, dT, or dC has been reported.^[37]^

**Figure 8:**
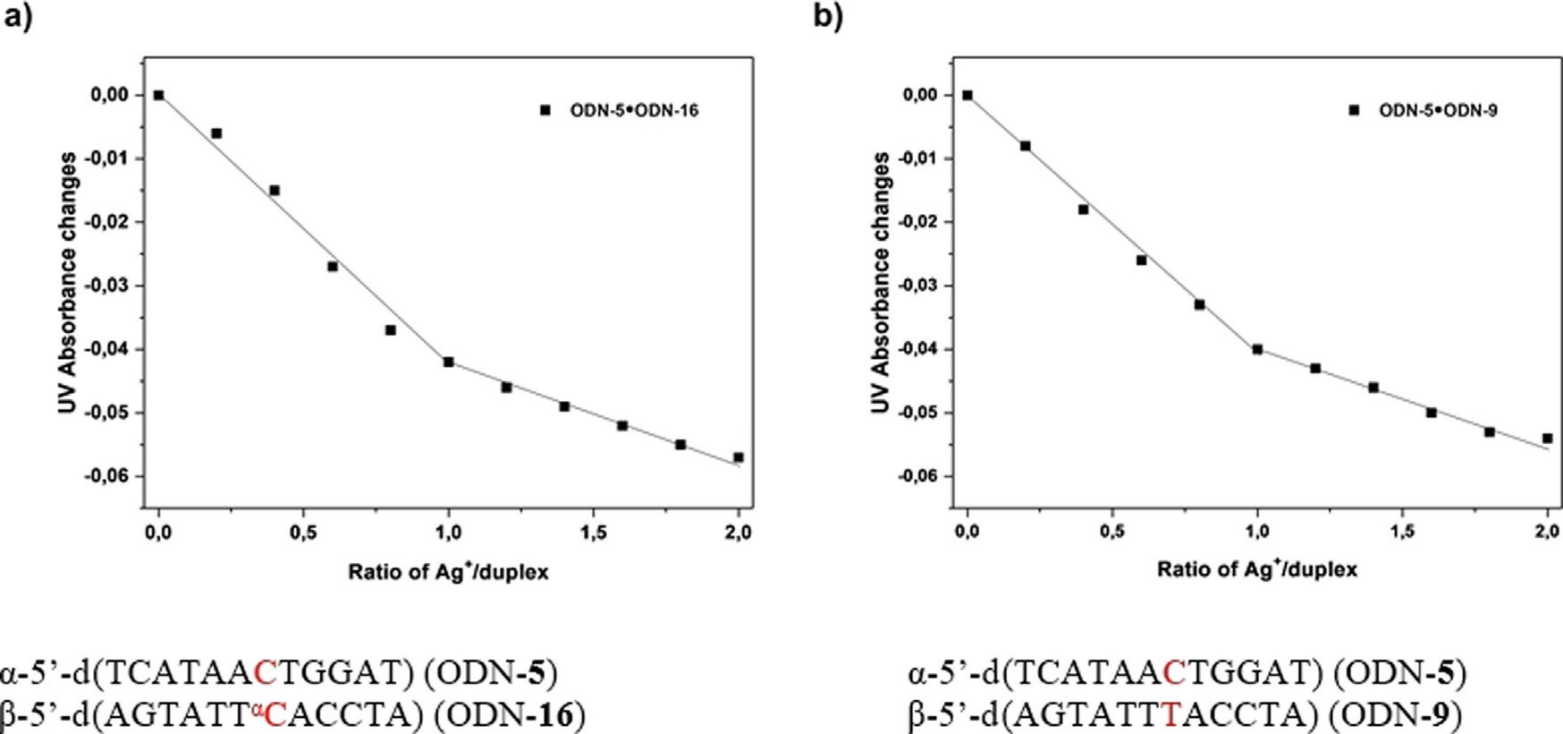
Titration graphs displaying the Ag^+^/duplex ratio vs. changes in UV absorbance measured at 260 nm for a) duplex ODN‐**5⋅**ODN‐**16** and b) ODN‐**5⋅**ODN‐**9**. All measurements were performed with 5 μm+5 μm single‐strand concentrations in 100 mm NaOAc and 10 mm Mg(OAc)_2_ (pH 7.4).

According to the findings on silver ion binding in homochiral DNA duplexes and the observation of metal‐mediated base pairing in parallel DNA,^[38]^ we anticipated that heterochiral DNA formed by complementary α‐d/β‐d strands would be capable of forming silver‐mediated base pairs when containing mismatches. Consequently, the formation of silver‐mediated base pairs involving α‐dC opposite dC, dT, dA, and α‐dC (β strand) was investigated in heterochiral duplexes. The *T*_m_ values were compared with those for β‐dC opposite the canonical bases (Table 6). For comparison, fully matched duplexes are also shown. From stoichiometric titration experiments, a ratio of one silver ion per duplex was confirmed (Figure 8).

**Table 6:** *T*_m_ values of homo‐ and heterochiral DNA duplexes constructed from complementary strands with α‐d or β‐d configuration in the presence and absence of silver ions.^[a]^

Duplexes	*T*_m_ [°C]	*T*_m_ [°C] 1 Ag/ds (Δ*T*_m_ [°C])	*T*_m_ [°C] 2 Ag/ds (Δ*T*_m_ [°C])	Duplexes	*T*_m_ [°C]	*T*_m_ [°C] 1 Ag/ds (Δ*T*_m_ [°C])	*T*_m_ [°C] 2 Ag/ds (Δ*T*_m_ [°C])
α‐5′‐d(TCATAACTGGAT) (ODN‐**5**) β‐5′‐d(AGTATTGACCTA) (ODN‐**2**)	41.0	40.0 (−1)	39.0 (−2)	β‐5′‐d(TAGGTCAATACT) (ODN‐**1**) β‐3′‐d(ATCCAGTTATGA) (ODN‐**2**)	47.0	48.0 (+1)	n.m.
				β‐5′‐d(TAGGTAAATACT) (ODN‐**23**) β‐3′‐d(ATCCATTTATGA) (ODN‐**24**)	42.0	42.0 (±0)	42.0 (±0)
α‐5′‐d(TCATAACTGGAT) (ODN‐**5**) β‐5′‐d(^7^A^7^GT^7^ATT^7^G^7^ACCT^7^A) (ODN‐**10**)	36.0	35.0 (−1)	35.0 (−1)	β‐5′‐d(TAGGTCAATACT) (ODN‐**1**) β‐3′‐d(^7^ATCC^7^A^7^GTT^7^AT^7^G^7^A) (ODN‐**10**)	43.0	45.0 (+2)	46.0 (+3)
α‐5′‐d(TCATAACTGGAT) (ODN‐**5**) β‐5′‐d(AGTATTAACCTA) (ODN‐**8**)	<15	24.0 (+>9)	24.0 (+>9)	β‐5′‐d(TAGGTCAATACT) (ODN‐**1**) β‐3′‐d(ATCCAATTATGA) (ODN‐**8**)	25.0	29.0 (+4)	n.m.
α‐5′‐d(TCATAACTGGAT) (ODN‐**5**) β‐5′‐d(AGTATTTACCTA) (ODN‐**9**)	<15	23.0 (+>8)	23.0 (+>8)	β‐5′‐d(TAGGTCAATACT) (ODN‐**1**) β‐3′‐d(ATCCATTTATGA) (ODN‐**9**)	26.0	35.0 (+9)	n.m.
α‐5′‐d(TCATAACTGGAT) (ODN‐**5**) β‐5′‐d(AGTATTCACCTA) (ODN‐**7**)	<15	28.0 (+>13)	28.0 (+>13)	β‐5′‐d(TAGGTCAATACT) (ODN‐**1**) β‐3′‐d(ATCCACTTATGA) (ODN‐**7**)	27.0	34.0 (+7)	n.m.
α‐5′‐d(TAGGTCAATACT) (ODN‐**6**) β‐5′‐d(ATCCACTTATGA) (ODN‐**21**)	19.5	27.0 (+7.5)	n.m.				
α‐5′‐d(TCATAACTGGAT) (ODN‐**5**) β‐5′‐d(AGTATT^α^CACCTA) (ODN‐**16**)	26.0	37.0 (+11)	37.0 (+11)	β‐5′‐d(TAGGTCAATACT) (ODN‐**1**) β‐3′‐d(ATCCA^α^CTTATGA) (ODN‐**16**)	30.0	44.0 (+14)	n.m.
α‐5′‐d(TAGGTCAATACT) (ODN‐**6**) β‐5′‐d(ATCCA^α^CTTATGA) (ODN‐**22**)	24.5	30.0 (+5.5)	n.m.				
α‐5′‐d(TCATAACTGGAT) (ODN‐**5**) β‐5′‐d(AGTATT1ACCTA) (ODN‐**19**)	<15	21.0	20.0	β‐5′‐d(TAGGTCAATACT) (ODN‐**1**) β‐3′‐d(ATCCA1TTATGA) (ODN‐**19**)	27.0	30.0 (+3)	31.0 (+4)
α‐5′‐d(TCATAACTGGAT) (ODN‐**5**) β‐5′‐d(AGTATT2ACCTA) (ODN‐**20**)	25.0	36.0 (+11)	36.0 (+11)	β‐5′‐d(TAGGTCAATACT) (ODN‐**1**) β‐3′‐d(ATCCA2TTATGA) (ODN‐**20**)	29.0	40.0 (+11)	42.0 (+13)
α‐5′‐d(TCATAACTGGAT) (ODN‐**5**) β‐5′‐d(ACTATTCACCTA) (ODN‐**17**)	<15	<15	<15	β‐5′‐d(TAGGTCAATACT) (ODN‐**1**) β‐3′‐d(ATCCACTTATCA) (ODN‐**17**)	<20	n.m.	27.0 (+>7)
α‐5′‐d(TCATAACTGGAT) (ODN‐**5**) β‐5′‐d(A^α^CTATT^α^CACCTA) (ODN‐**18**)	<15	31.0 (+>16)	31.0 (+>16)	β‐5′‐d(TAGGTCAATACT) (ODN‐**1**) β‐3′‐d(ATCCA^α^CTTAT^α^CA) (ODN‐**18**)	22.0	n.m.	38.0 (+16)

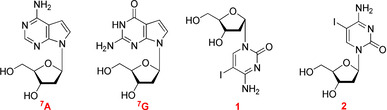							

[a] Measured at 260 nm with 5 μm+5 μm single‐strand concentrations at a heating rate of 1.0 °C min^−1^ in 100 mm NaOAc and 10 mm Mg(OAc)_2_ (pH 7.4) in the presence of various concentrations of AgNO_3_ (0, 1, and 2 silver ions per duplex). *T*_m_ values were calculated from the cooling curves using the Meltwin 3.0 program.^[25]^ ^7^A corresponds to 7‐deaza‐2′‐deoxyadenosine, ^7^G corresponds to 7‐deaza‐2′‐deoxyguanosine, 1 corresponds to 5‐iodo‐2′‐deoxycytidine, and 2 corresponds to α‐5‐iodo‐2′‐deoxycytidine.^[23]^ ds corresponds to double‐stranded DNA. n.m. refers to not measured.

**Figure 9:**
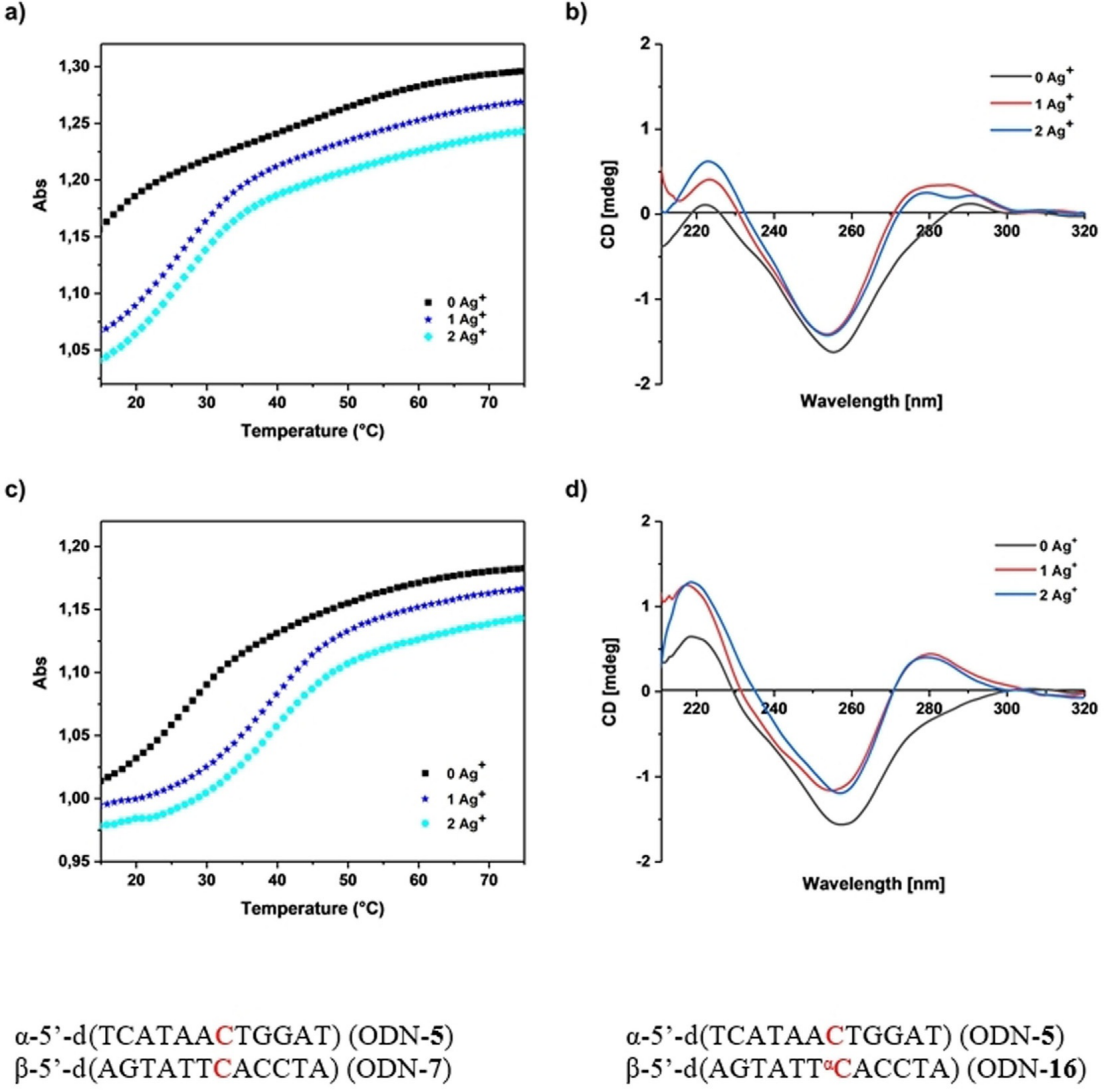
Melting curves of duplexes a) ODN‐**5⋅**ODN‐**7** and c) ODN‐**5⋅**ODN‐**16**. Temperature‐dependent CD spectra of duplexes b) ODN‐**5⋅**ODN‐**7** and d) ODN‐**5⋅**ODN‐**16**. Measured with 5 μm+5 μm single‐strand concentrations in 100 mm NaOAc and 10 mm Mg(OAc)_2_ (pH 7.4) at 260 nm in the presence of various concentrations of Ag^+^ (0, 1, and 2 silver ions/duplex). The cell path length of the cuvette used to record the UV spectra was 1.0 cm and 0.2 cm for the CD spectra.

The melting curves and CD spectra of these duplexes are shown in Figure 9 and Figures S25–S29 in the Supporting Information. The melting curves of all the duplexes with mismatches show a strong initial decrease in absorbance after addition of silver (Figure 9 a,c). This is because a portion of the oligonucleotides containing mismatches exist as single strands in solution before the addition of silver ions. After the addition of silver ions, silver‐mediated base pairs are formed between mismatches and Watson–Crick base pairs between matches. This leads to substantial hypochromicity. The CD spectra recorded with increasing amounts of silver ions (0–2 Ag^+^) indicate the conversion from single‐stranded oligonucleotides into stable duplexes. The positive lobe at 278 nm appears to demonstrate the B‐DNA structure (Figure 9 b,d).

The following conclusions can be drawn from the data presented in Table 6.

1. The most stable duplex formed contains the silver‐mediated base pair of α‐dC with α‐dC (ODN‐**5⋅**ODN‐**16**; *T*_m_=37 °C and Δ*T*_m_=11 °C). The stability is in the range of the fully matched duplex ODN‐**5⋅**ODN‐**2** (*T*_m_=41 °C). Addition of silver ions to the duplex ODN‐**5⋅**ODN‐**18** with two α‐dC**⋅**α‐dC mismatches also leads to a stable duplex (*T*_m_=31 °C), whereas no duplex formation is observed when two α‐dC**⋅**dC base pairs are present.
2. The heterochiral duplex with α‐5‐iodo‐2′‐deoxycytidine–Ag^+^–α‐dC shows similar stabilization (*T*_m_=36 °C and Δ*T*_m_=11 °C) to the α‐dC**⋅**α‐dC pair. Thus, 5‐iodo substituents are well accommodated in heterochiral α/β DNA with parallel chain orientation.
3. The reversed duplex to ODN‐**5⋅**ODN‐**16**, namely ODN‐**6⋅**ODN‐**22**, shows a lower *T*_m_ increase (Δ*T*_m_=5.5 °C) after silver addition. Probably, the geometry of the silver‐mediated α‐dC**⋅**α‐dC base pair in the “original” duplex is more favored than the geometry of the silver pair in the reversed duplex.
4. A significant influence of nearest neighbors on all silver‐mediated base pairs has to be considered, leading to sequence‐dependent differences in the *T*_m_ values.
5. Strong duplex stabilization is observed when silver ions are added to oligonucleotides containing α‐dC opposite dC, dA, and dT. The *T*_m_ increase is in the same range or higher than those reported for the corresponding β‐dC mismatches (Table 6).
6. Most strikingly, heterochiral parallel α/β DNA duplexes show almost the same properties with respect to silver‐mediated base pairing as the corresponding homochiral antiparallel β/β duplexes. Thus, this system can be expected to be a bioorthogonal pairing system. From this, it can be concluded that the structures of silver‐mediated base pairs are similar for specific nucleobase interactions in α/β and β/β duplexes. Possible base pairs are shown in Figure 10
  **Figure 10:**
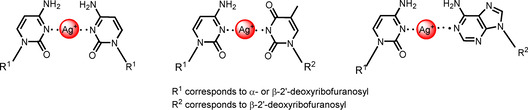
Proposed structures for silver‐mediated base pairs.
  .

### Formation of heterochiral DNA–silver complexes at high silver ion concentration

A strong excess of silver ions with respect to DNA base pairs leads to the formation of unusual stable silver complexes. The X‐ray analysis of a 12‐mer duplex showed the formation of a metal–DNA nanowire with an uninterrupted one‐dimensional silver array.^[30]^ This wire consists of silver‐mediated homo pairs of dT**⋅**dT, dG**⋅**dG, and dC**⋅**dC instead of Watson–Crick base pairs. Other complex structures exist that rely on the sequence and amount of silver ions added to the DNA duplexes.[^30^, ^34b^, ^39^, ^40^, ^41^] Argentophilic interactions have also to be considered.^[42]^

**Figure 11:**
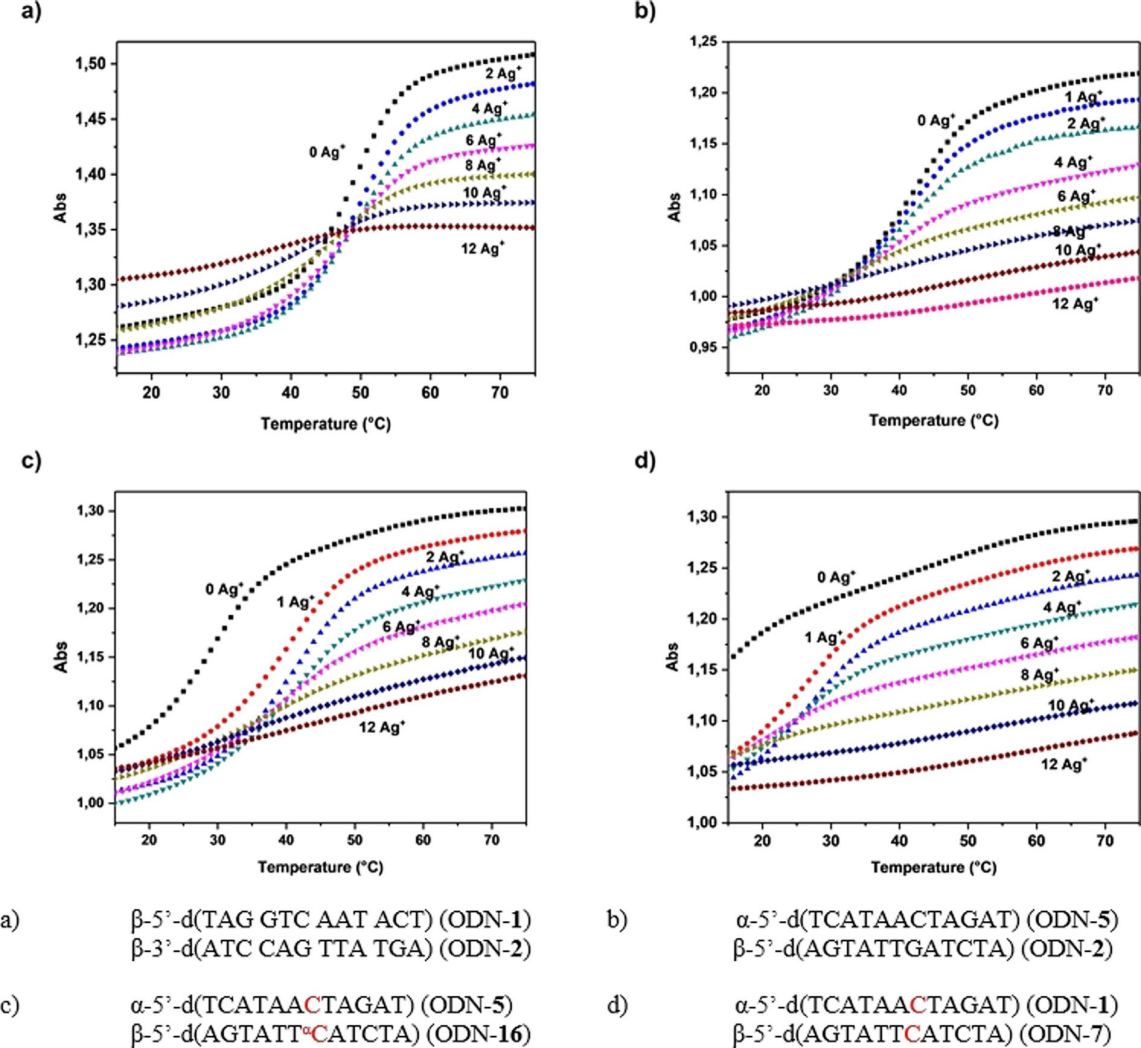
Thermal denaturation experiments performed at a 5 μm+5 μm single‐strand concentration in 100 mm NaOAc and 10 mm Mg(OAc)_2_ (pH 7.4), monitoring at 260 nm in the presence of various concentrations of Ag^+^ (0, 1, 2, 4, 6, 8, 10, and 12 silver ions per duplex): a) ODN‐**1⋅**ODN‐**2**, b) ODN‐**5⋅**ODN‐**2**, c) ODN‐**5⋅**ODN‐**16**, and d) ODN‐**1⋅**ODN‐**7**.

Based on the observations of homochiral 12‐mer duplexes that can ligate more silver ions than the mismatches that they contain,[^22^, ^34b^] we anticipated that heterochiral duplexes can form silver‐mediated base pairs between natural bases to form complexes that show similar behavior to that of canonical DNA. To this end, melting curves were measured for increasing equivalents of silver ions from 1 to 12. Heating and cooling cycles were recorded, and normalized melting curves were calculated. Figure 11 b–d demonstrates the situation for heterochiral duplexes, whereas Figure 11 a shows the UV changes for the homochiral 12‐mer duplex ODN‐**1⋅**ODN‐**2**, which has already been reported.[^22^, ^34b^]

The UV melting profiles of the heterochiral duplexes containing a dG**⋅**dC base pair or α‐dC**⋅**dC or α‐dC**⋅**α‐dC mismatches show that a change in the duplex structure also takes place in these cases. The flattening of the curve and absence of a melting point at high silver concentration are the result of the formation of a DNA–silver complex that contains 12 silver ions per duplex, one silver ion per base pair. This complex is very stable and does not show sigmoidal melting in the measured temperature range. Several heating and cooling cycles had to be performed to obtain identical melting profiles as the first heating cycle showed hysteresis at high silver ion concentration. Furthermore, the melting curves of the β/β complex ODN‐**1⋅**ODN‐**2** show an isosbestic point (Figure 11 a), which is not the case for the heterochiral duplexes (Figure 11 b–d). For α/β duplexes the melting curve becomes a straight line already after addition of eight silver ions, whereas 12 silver ions are required for the homochiral complex to obtain an almost straight line.. The addition of more than 12 silver ions per complex leads to a straight‐line melting profile in both cases (see Figure S30 a,b in the Supporting Information). Thus, smaller amounts of silver are required to turn heterochiral complexes into another species. It can be assumed that this is a result of the different stabilities of heterochiral Watson–Crick and silver‐mediated base pairs compared with homochiral ones. These findings confirm that there are strong similarities between silver‐mediated parallel α/β and antiparallel β/β duplexes.

**Figure 12:**
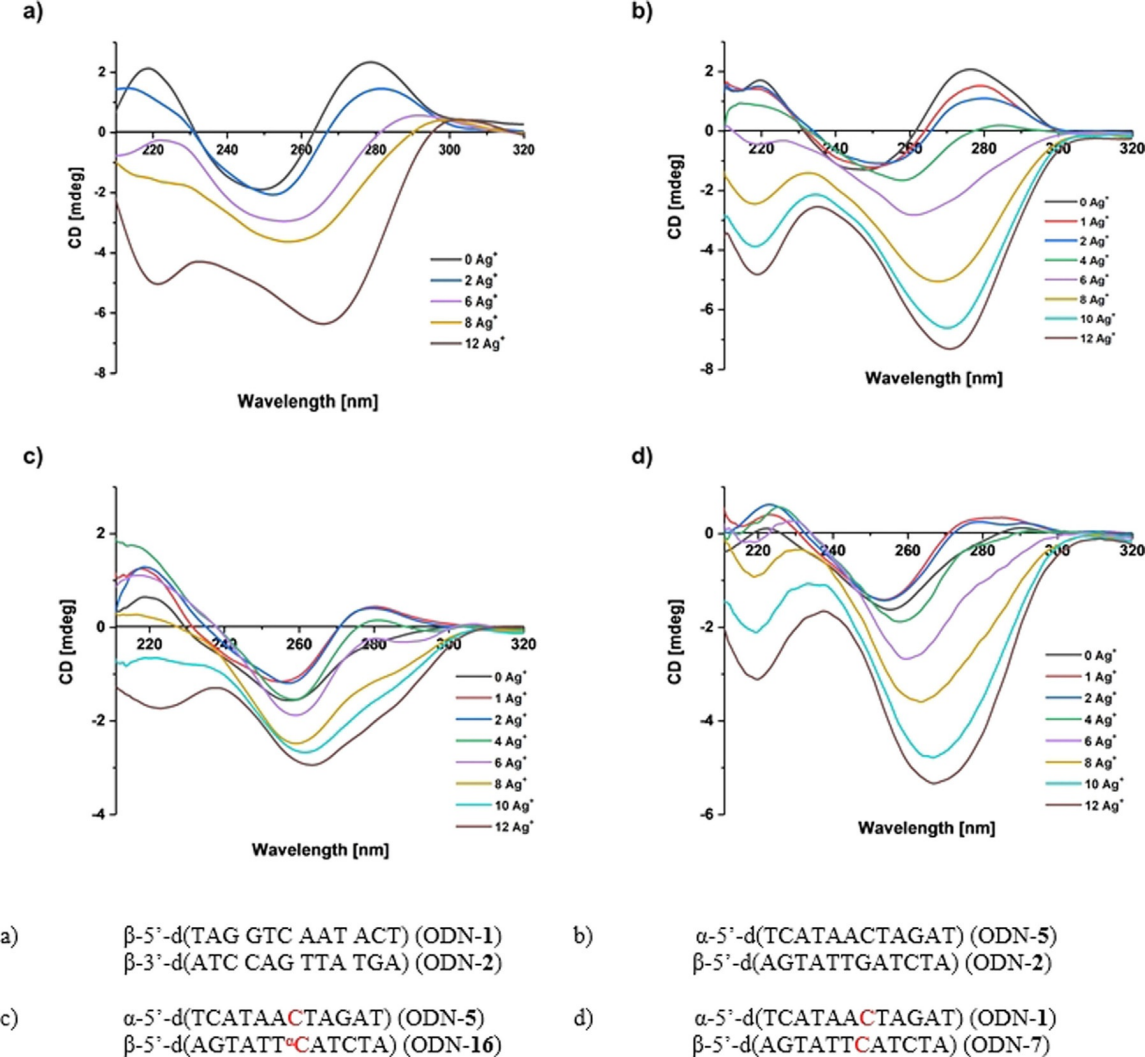
CD spectra of the duplexes a) ODN‐**1⋅**ODN‐**2**, b) ODN‐**5⋅**ODN‐**2**, c) ODN‐**5⋅**ODN‐**16**, and d) ODN‐**1⋅**ODN‐**7**. The CD spectra were recorded with 5 μm+5 μm single‐strand concentrations in 100 mm NaOAc and 10 mm Mg(OAc)_2_ (pH 7.4), monitoring at 260 nm in the presence of various concentrations of Ag^+^ (0, 1, 4, 6, 8, 10, and 12 silver ions per duplex). The cell path length of the cuvette used to record the CD spectra was 0.2 mm.

Next, CD spectra were measured to visualize the changes in the helical structure of DNA (Figure 12). Hetero‐ and homochiral silver‐free duplexes without mismatches show conventional CD spectra with a positive lobe at around 275 nm and a negative band near 245 nm (Figure 12 a,b). The CD spectra of heterochiral duplexes with α‐dC**⋅**dC or α‐dC**⋅**α‐dC mismatches show single strands at 25 °C (Figure 12 c,d).

The addition of silver ions leads to dramatically altered CD spectra. For both the heterochiral duplex ODN‐**5⋅**ODN‐**2** and the homochiral duplex ODN‐**1⋅**ODN‐**2**, the positive lobes at 278 nm disappear and the negative lobes at 248 nm are bathochromically shifted (ca. 30 nm) with a strongly increased negative amplitude (Figure 12 a,b). Similar shapes of the CD curves were obtained for both kinds of duplexes after the addition of 12 silver ions. The same is true for the heterochiral duplexes ODN**‐1⋅**ODN**‐7** (α‐dC**⋅**dC mismatch) and ODN**‐5⋅**ODN**‐16** (α‐dC**⋅**α‐dC; Figure 12 c,d). The addition of more than 12 silver ions per duplex did not lead to further significant changes in the CD spectra in all cases (see Figure S30 c,d in the Supporting Information). This indicates that heterochiral α/β duplexes behave like β/β duplexes in the presence of large amounts of silver ions. The typical B‐DNA structure consisting of silver‐free Watson–Crick base pairs is converted into a complex with silver‐mediated base pairs. Thus, the particular structure and type of silver‐mediated base pairs of each of these duplexes seem to depend mainly on the individual oligonucleotide sequence but not on the chirality (hetero‐ or homochiral) of the duplexes. These findings confirm that there are strong similarities between silver‐mediated parallel α/β and antiparallel β/β duplexes.

## Conclusions and Outlook

In this study, the stability of hydrogen‐bonded and silver‐mediated base pairs as well as the impact of 7‐deazapurines in place of purines have been investigated for heterochiral DNA displaying a parallel chain orientation. First, 12‐mer duplexes were constructed from single‐stranded oligonucleotides with one strand in the β‐d configuration and the other in the α‐d configuration. To this end, phosphoramidites with nucleoside residues with α‐d and β‐d configurations were assembled to form oligonucleotides. From the *T*_m_ values and thermodynamic data it is evident that the thermal stability of heterochiral DNA is similar to that of homochiral DNA. Mismatch discrimination was better in heterochiral DNA. This might be advantageous for the detection of genetic errors in diagnostic assays.

When all the purine bases of the β strand were replaced by 7‐deazapurines stable duplexes were formed. This is chemical proof for Watson–Crick base pairing and illustrates that nucleobases with modified skeletons are well accommodated in heterochiral DNA. In addition to 12‐mer duplexes, heterochiral 22‐mers, including DNA**⋅**DNA and DNA**⋅**RNA hybrids, were prepared. The DNA**⋅**RNA hybrids showed hysteresis in heterochiral hybrids, as was observed for homochiral counterparts. The CD spectra of single strands with α‐d configuration display a shape that is almost mirror‐like to the spectra of β strands, whereas the CD spectra of hydrogen‐bonded heterochiral double helices show B‐type structures almost indistinguishable from natural DNA.

When cytosine faces cytosine, 5‐iodocytosine (**1** or **2**), thymine, or adenine in heterochiral DNA silver‐mediated base pairs are formed. The most stable silver‐mediated pair is that with two cytosine nucleosides in the α‐d configuration, whereas the most stable cytosine pair in homochiral DNA is formed between anomeric cytosine nucleoside residues.

**Figure 13:**
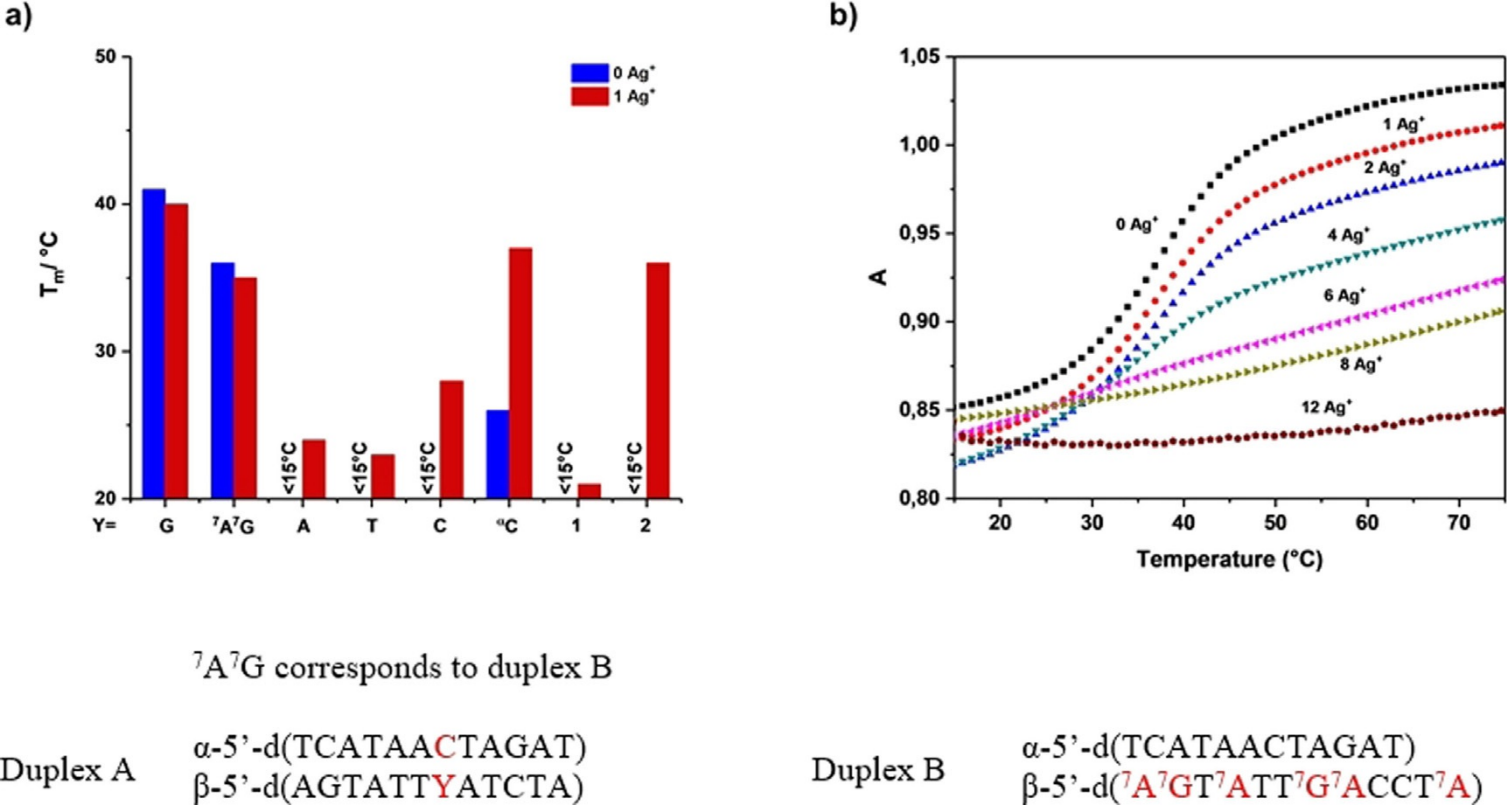
a) Bar diagram showing the *T*_m_ values of heterochiral DNA duplexes. Measurements were performed in the absence (blue) and presence of one silver ion (red), according to Table 6. b) Melting profiles of duplex B with increasing amounts of silver ions (0–12 silver ions per duplex).

When the sequences of both strands were reversed, slight changes in the structure and stability were observed in hetero‐ and homochiral DNA. The bar diagram in Figure 13 a illustrates the situation. The blue bars indicate *T*_m_ values in the absence of silver, the red bars in the presence of silver. As the number of silver ions increases from 2 to 12, heterochiral DNA binds more silver ions and forms a nonmelting complex (Figure 13 b), a phenomenon that is also observed in homochiral DNA.

From this and related studies,^[43]^ the question arises as to why nature chose the antiparallel instead of the parallel strand orientation for double‐helical DNA. Antiparallel strand orientation leads to serious problems during replication. Although the leading strand of DNA can incorporate 5′‐phosphates continuously, the lagging strand cannot. RNA primers are required and Okazaki fragments are formed, which are then ligated afterwards. Heterochiral DNA with parallel strands would not have this problem. Enzymatic polymerization can proceed in both strands in the same direction continuously. However, as shown for heterochiral DNA, the system requires eight building blocks, four with the α‐d configuration and four with the β‐d configuration. Apparently, this was a more serious problem for evolution than those arising in the replication fork. Nevertheless, chemical polymerization of α‐nucleoside phosphates on β‐d oligonucleotide templates or β nucleotides on α‐oligonucleotide templates should proceed efficiently. Furthermore, heterochiral DNA shows properties, for example, stability against enzymatic degradation, that makes its application valuable for the construction of DNA‐based materials and nanodevices, for applications as sensors, and for diagnostic purposes.

## Experimental Section

### General methods and materials

All chemicals and solvents were of laboratory grade as obtained from commercial suppliers and were used without further purification. Reversed‐phase HPLC was carried out by using a HITACHI 655A‐12 liquid chromatograph connected to L6200A intelligent pump and 655A variable‐wavelength UV monitor with a 4×250 mm RP‐18 (10 μm) LiChrospher 100 column. The molecular masses of the oligonucleotides were determined by MALDI‐TOF MS on a Bruker Autoflex Speed spectrometer in linear positive mode with 3‐hydroxypicolinic acid as matrix. The thermal melting curves were measured with an Agilent Technologies Cary 100 Bio UV‐vis spectrophotometer equipped with a thermoelectric controller. The temperature was measured continuously in the reference cell using a Pt‐100 resistor at a heating rate of 1 °C min^−1^. The *T*_m_ values were determined from the melting curves using the software Meltwin (version 3.0).^[25]^ CD spectra were recorded at 25 °C on a Jasco J‐815 spectrometer.

### Oligonucleotide syntheses and characterization

Solid‐phase oligonucleotide syntheses were performed on an ABI 392‐08 synthesizer on a 1 μmol scale (trityl‐on mode) employing the phosphoramidites of α‐dC, α‐dA, α‐dT, and α‐dG, the building blocks α‐ and β‐5‐iodocytidine, 7‐deaza‐2′‐deoxyadenosine, and 7‐deaza‐2′‐deoxyguanosine as well as the standard building blocks with an average coupling yield of over 95 %. After cleavage from the solid support, the oligonucleotides were deprotected in 28 % aqueous ammonia at 55 °C for 2 h. The dimethoxytrityl‐containing oligonucleotides were purified by reversed‐phase HPLC (RP‐18) with gradient elution using detection at 260 nm: solution A: MeCN; solution B: 0.1 m (Et_3_NH)OAc (pH 7.0)/MeCN, 95:5; gradient I: 0–3 min 10–15 % A in B, 3–15 min 15–50 % A in B; flow rate: 0.7 mL min^−1^. The purified “trityl‐on” oligonucleotides were treated with 2.5 % CHCl_2_COOH/CH_2_Cl_2_ for 2 min at 0 °C to remove the 4,4′‐dimethoxytrityl residues. The detritylated oligomers were purified again by reversed‐phase HPLC with gradient elution: Gradient II: 0–20 min 0–20 % A in B, 20–25 min 20 % A in B; flow rate: 0.7 mL min^−1^. The oligonucleotides were desalted on a reversed‐phase column (RP‐18) using water for elution of the salt, and the oligonucleotides were eluted with H_2_O/CH_3_OH (2:3). The oligonucleotides were lyophilized on a Speed‐Vac evaporator to yield colorless solids, which were then frozen at −24 °C. The purity of all oligonucleotides was confirmed by RP‐18 HPLC (see Figures S2–S23 in the Supporting Information) and MALDI‐TOF MS (Table 1). The extinction coefficients *ϵ*_260_ (H_2_O) of the nucleosides are as follows: dA 15 400, dG 11 700, dT 8800, dC 7300, α‐dC 7300, α‐dA 15 400, α‐dT 8800, α‐dG 11 700, ^7^A 8100,^[22]^ ^7^G 13 400,^[44]^ 5‐I‐dC 3100 (MeOH),^[23]^ and α‐5‐I‐dC 3400 dm^3^ mol^−1^ cm^−1^ (MeOH).^[23]^ The extinction coefficients of the oligonucleotides were calculated from the sum of the extinction coefficients of the nucleoside constituents considering the hypochromic changes for the particular single strands. Oligoribonucleotide microRNA (miRNA‐122; ODN‐**15**) was provided by a commercial supplier.

### General procedure for the stoichiometric titration experiments

A AgNO_3_ stock solution (2 μL) was added stepwise to solution containing 2.5 μm+2.5 μm single‐stranded oligonucleotides in 100 mm NaOAc and 10 mm Mg(OAc)_2_ buffer (1 mL, pH 7.4) to obtain 0.2 equiv of silver ions/duplex. Then, in a stepwise manner, 2 μL of the AgNO_3_ stock solution was added. After each addition of the stock solution, the mixture was equilibrated for 5 min at 20 °C. The UV/Vis absorption spectra were then recorded in the range 220–450 nm. The changes in absorbance at selected wavelengths were plotted versus the silver ions/duplex ratio to establish the stoichiometry.

## Conflict of interest

The authors declare no conflict of interest.

## Supporting information

As a service to our authors and readers, this journal provides supporting information supplied by the authors. Such materials are peer reviewed and may be re‐organized for online delivery, but are not copy‐edited or typeset. Technical support issues arising from supporting information (other than missing files) should be addressed to the authors.

Supplementary
