# Supplementary material for: Heterochiral DNA with Complementary Strands with α‐d and β‐d Configurations: Hydrogen‐Bonded and Silver‐Mediated Base Pairs with Impact of 7‐Deazapurines Replacing Purines
Source: Chemistry. 2020 Sep 30;26(61):13973–89. doi: 10.1002/chem.202002765 (PMC7702046; doi:10.1002/chem.202002765)
Supplement: Supplementary file 1 — Supplementary [file CHEM-26-13973-s001.pdf]

Chemistry–A European Journal

Supporting Information

Heterochiral DNA with Complementary Strands with α-D and β-D Configurations: Hydrogen-Bonded and Silver-Mediated Base Pairs with Impact of 7-Deazapurines Replacing Purines

Yingying Chai,[a, b] Xiurong Guo,[a] Peter Leonard,[a] and Frank Seela*[a, c]

Table of Contents

Figure S1. Modified nucleosides used in this study S2

Table S1. T_m values and thermodynamic data of 22-mer duplexes S2

Reference S2

Figure S2-S23. Reversed HPLC profiles of purified oligonucleotides S3-S10

Figure S24. Melting profiles of heterochiral (α/β) oligonucleotide duplexes containing α-dC opposite the canonical bases (dG, dA, dC, dT) S11

Figure S25. Melting profiles of heterochiral (α/β) oligonucleotide duplexes containing α-dC opposite dG, dA, dC, dT in the absence and presence of silver ions S12

Figure S26. Melting profiles of heterochiral (α/β) oligonucleotide duplexes containing one or two dC/α-dC or α-dC/α-dC base pairs in the presence and absence of silver ions S13

Figure S27. Melting profiles of homochiral (β/β) and heterochiral (α/β) duplexes containing α-and β-5-iodo substituted dC in the presence and absence of silver ions S14

Figure S28. Melting profiles of homochiral (β/β) and heterochiral (α/β) duplexes containing 7-deaza-dA and 7-deaza-dG in in the presence and absence of silver ions S15

Figure S29. Melting profiles of reversed heterochiral (α/β) duplexes containing dC/α-dC or α-dC/α-dC base pairs in the presence and absence of silver ions S16

Figure S30. Melting profiles and CD spectra of duplexes ODN-**1**•ODN-**2** and ODN-**5**•ODN-**2** in the presence of various concentrations of Ag^+ (0-24 silver ions/duplex) S17

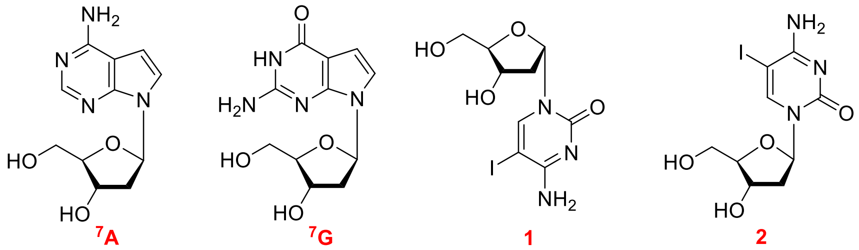

^{7}A ^{7}G   1   2

^{7}A: 7-Deaza-2'-deoxyadenosine; ^{7}G: 7-deaza-2'-deoxyguanosine; 1: α-5-iodo-2'-deoxycytidine; 2: 5-iodo-2'-deoxycytidine.

Figure S1. Modified nucleosides used in this study.

Table S1. T_m values and thermodynamic data of 22-mer oligonucleotide DNA/DNA and DNA/RNA duplexes.[a]

Duplexes	T_m [°C]	ΔH_{310} [kcal mol^{-1}]	ΔS_{310} [cal K^{-1} mol^{-1}]	ΔG_{310} [kcal mol^{-1}]
α-5'-d(ACCTCACACTGTTACCACAAAC) (ODN-**14**)<br>β-5'-d(TGGAGTGTGACAATGGTGTTTG) (ODN-**12**)	68	-155.8	-430.0	-22.4
α-5'-d(ACCTCACACTGTTACCACAAAC) (ODN-**14**)<br>β-5'-r(UGGAGUGUGACAAUGGUGUUUG) (ODN-**15**)	68	-176.5	-489.8	-24.6
β-5'-d(TGGAGTGTGACAATGGTGTTTG) (ODN-**12**)<br>β-3'-d(ACCTCACACTGTTACCACAAAC) (ODN-**13**)	71	-172.6	-474.8	-25.6
β-5'-r(UGGAGUGUGACAAUGGUGUUUG) (ODN-**15**)<br>β-3'-d(ACCTCACACTGTTACCACAAAC) (ODN-**13**)	73	-181.7	-497.7	-27.3

[a] Measured at 260 nm with 5 µM + 5 µM single-strand concentration at a heating rate of 1.0 °C/min in 0.1 M NaCl, 10mM $MgCl_2$, 10 mM Na-cacodylate. T_m values were calculated from the heating curves using the program Meltwin 3.0.[1]

[1] J. A. McDowell, D. H. Turner, *Biochemistry* **1996**, *35*, 14077–14089.

Reversed-phase (RP-18) HPLC profiles of oligonucleotides

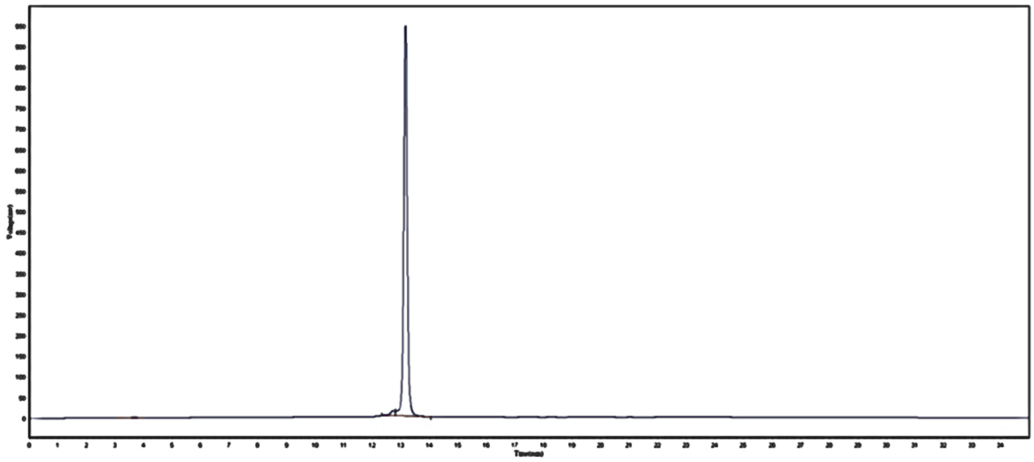

Figure S2. β-5’-d(TAGGTCAATACT) (ODN-**1**)

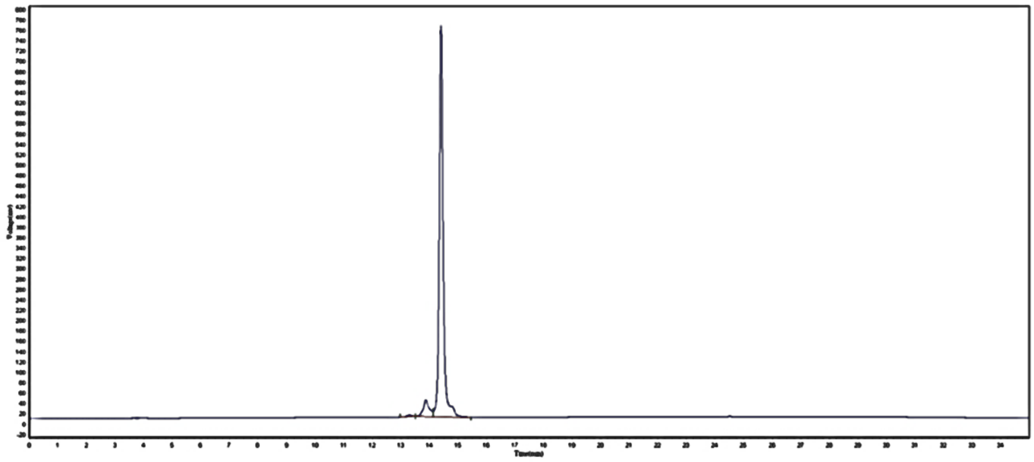

Figure S3. β-5’-d(AGTATTGACCTA) (ODN-**2**)

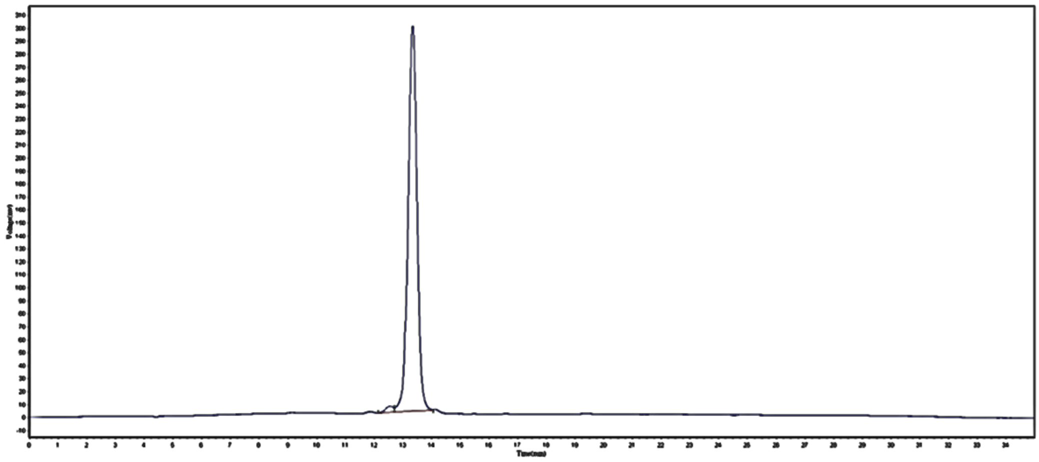

Figure S4. β-5’-d(ATCCAGTTATGA) (ODN-**3**)

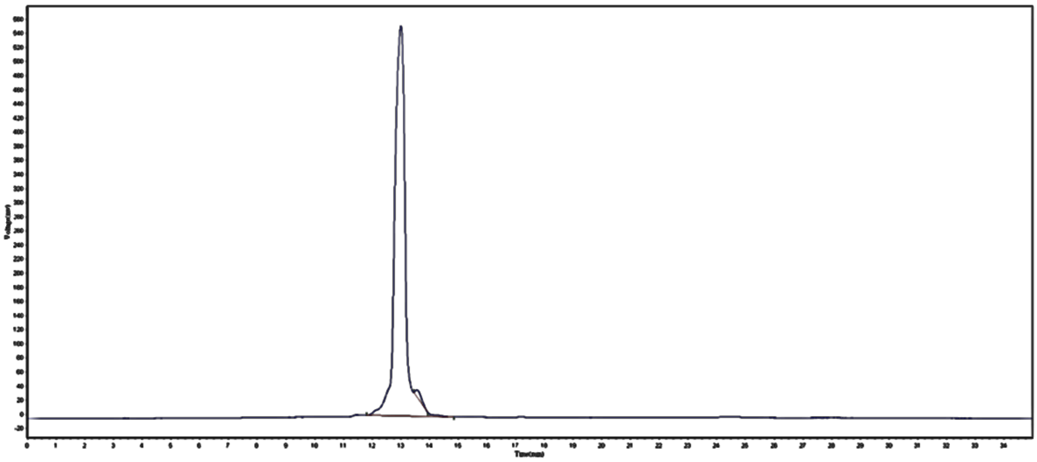

Figure S5. β-5’-d(TCATAACTGGAT) (ODN-**4**)

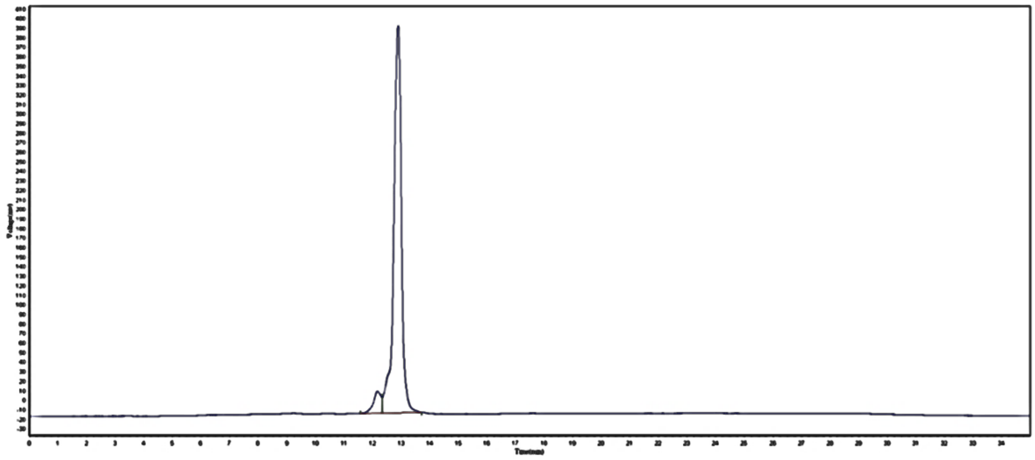

Figure S6. α-5’-d(TCATAACTGGAT) (ODN-**5**)

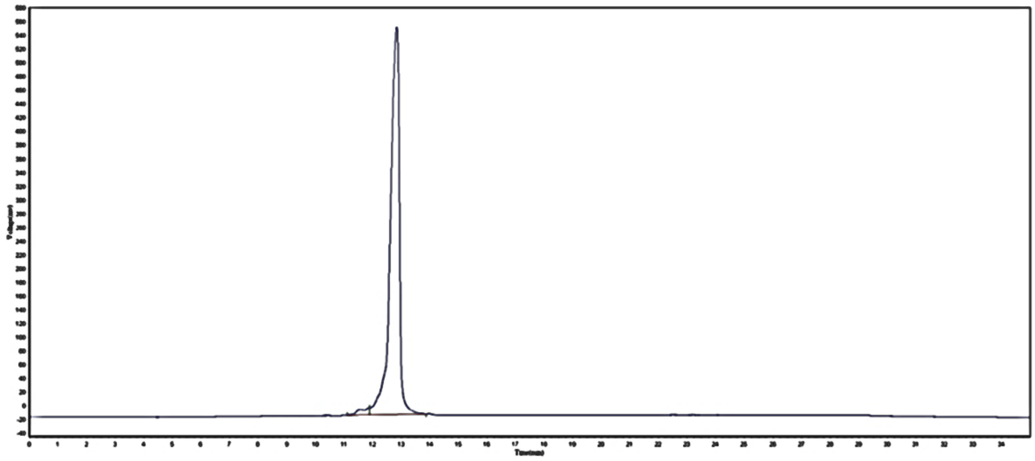

Figure S7. α-5’-d(TAGGTCAATACT) (ODN-**6**)

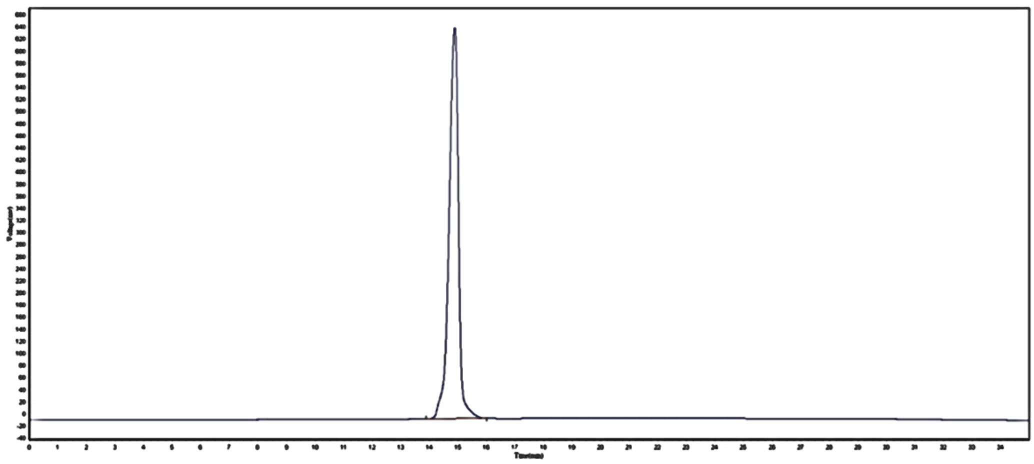

Figure S8. β-5’-d(AGTATTCACCTA) (ODN-**7**)

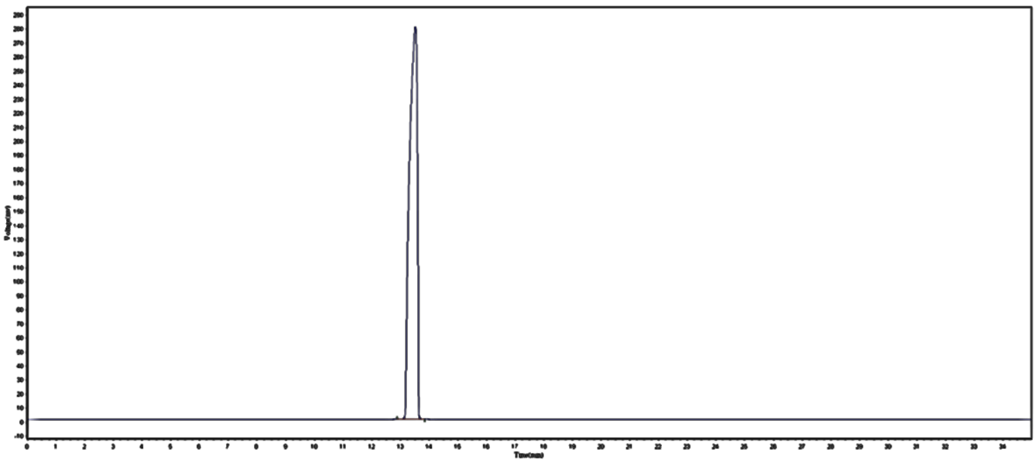

Figure S9. β-5’-d(AGTATTAACCTA) (ODN-**8**)

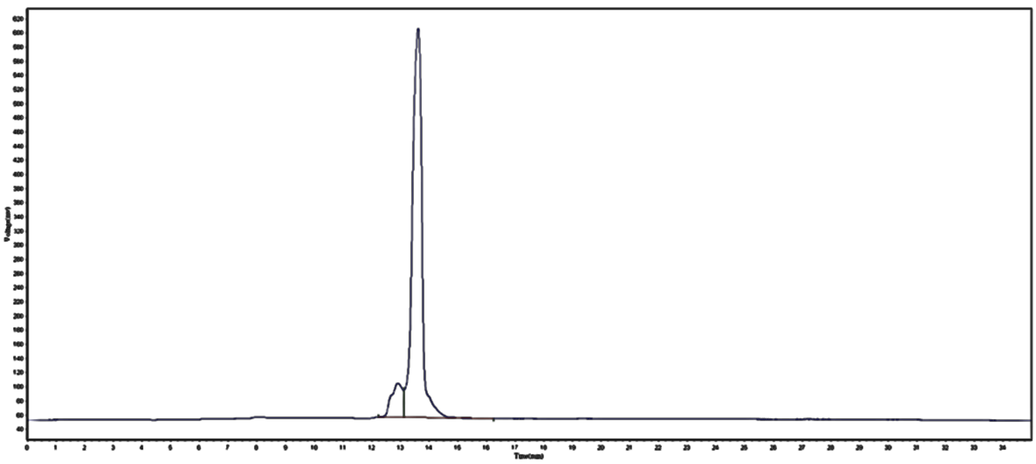

Figure S10. β-5’-d(AGTATTTACCTA) (ODN-**9**)

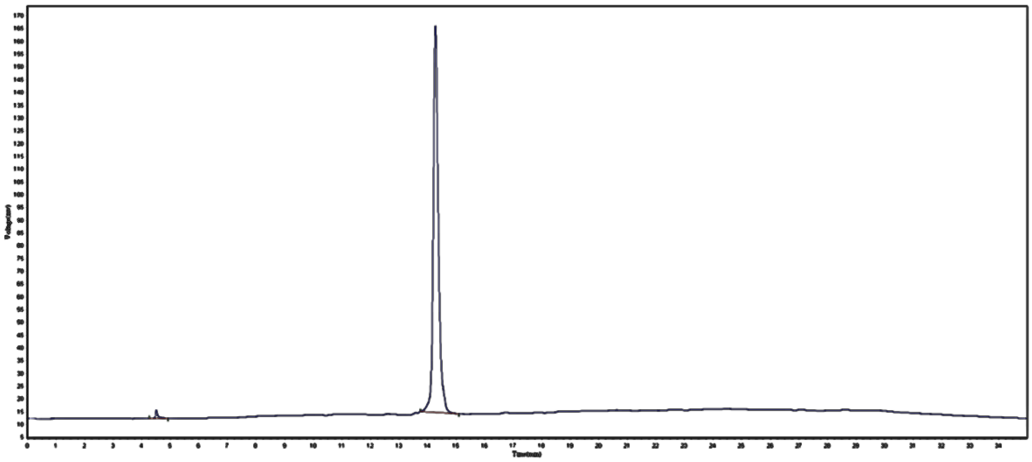

Figure S11. β-5'-d(^{7}A^{7}GT^{7}ATT^{7}G^{7}ACCT^{7}A) (ODN-**10**)

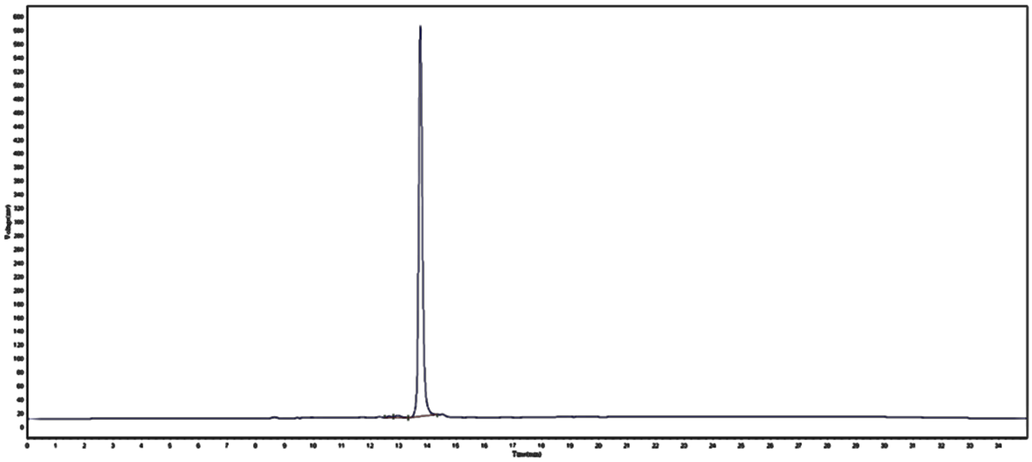

Figure S12. β-5'-d(T^{7}A^{7}G^{7}GTC^{7}A^{7}AT^{7}ACT) (ODN-**11**)

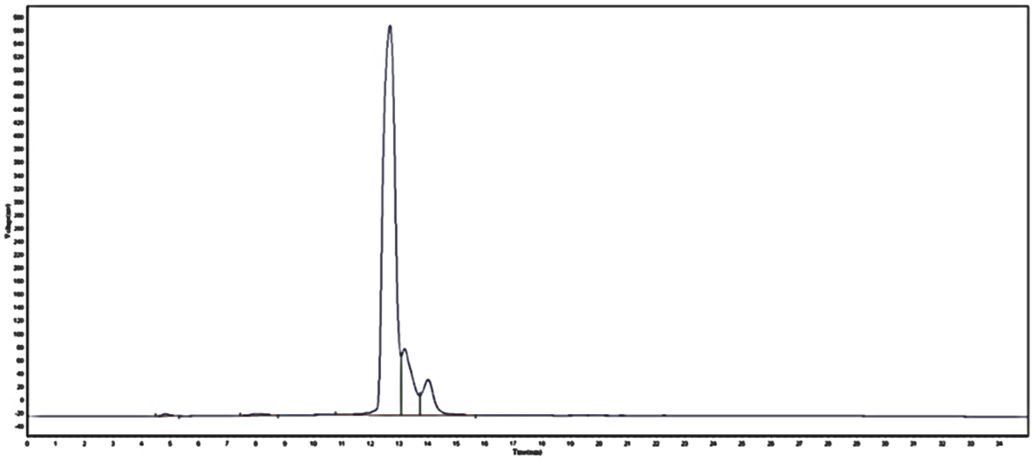

Figure S13. β-5'-d(TGGAGTGTGACAATGGTGTTTG) (ODN-**12**)

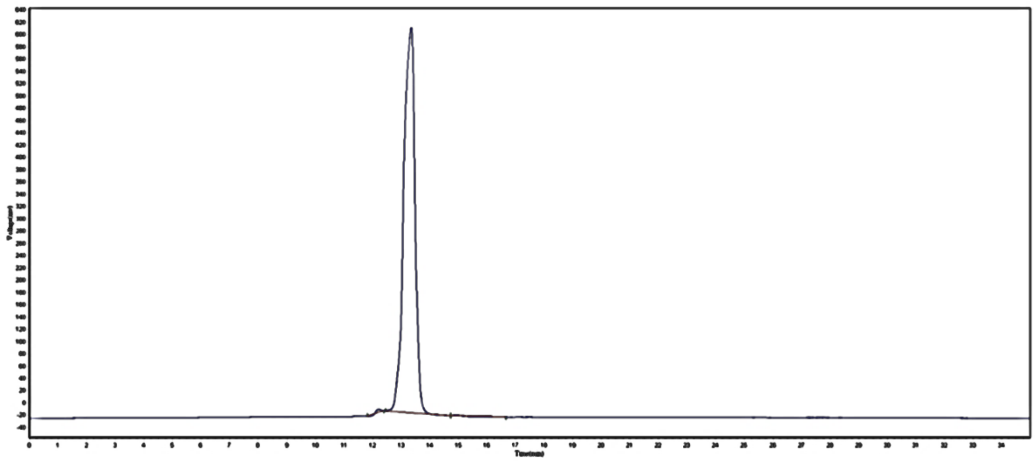

Figure 14. β-5’-d(CAAACACCATCACTCCATGTCA) (ODN-**13**)

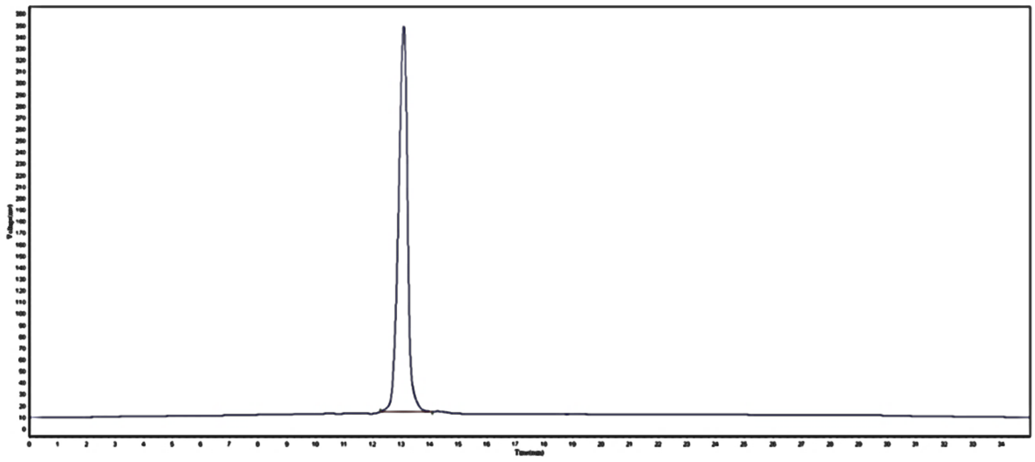

Figure S15. α-5’-d(ACCTCACACTGTTACCACAAAC) (ODN-**14**)

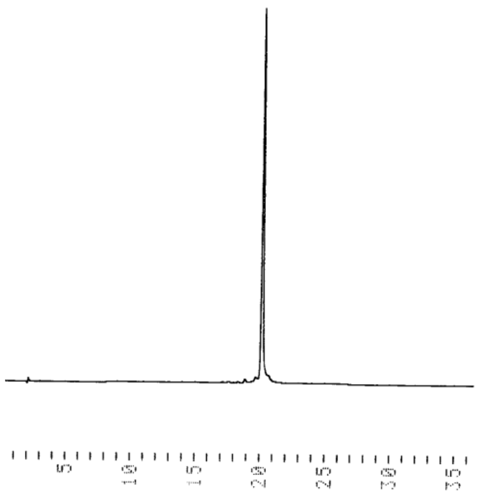

Figure S16. β-5’-r(UGGAGUGUGACAAUGGUGUUUG) (ODN-**15**)

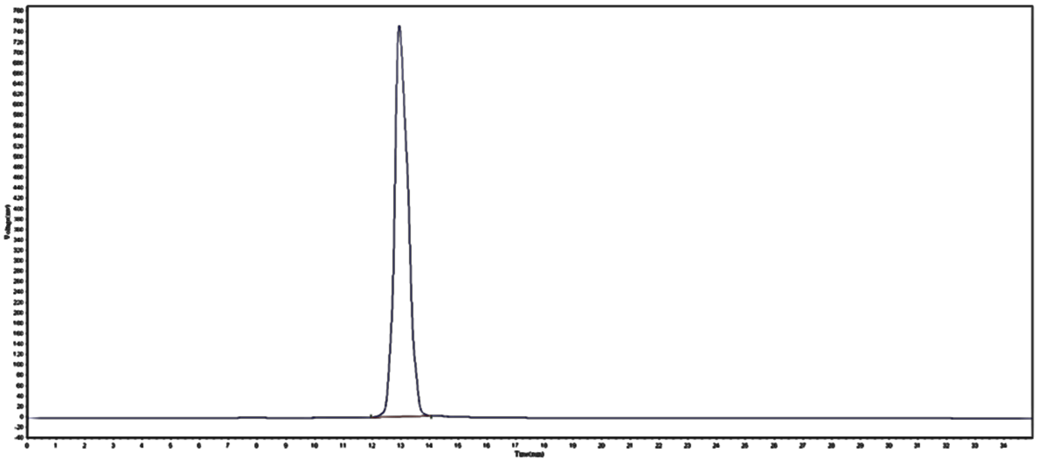

Figure S17. β-5’-d(AGTATT$^{\alpha}$CACCTA) (ODN-**16**)

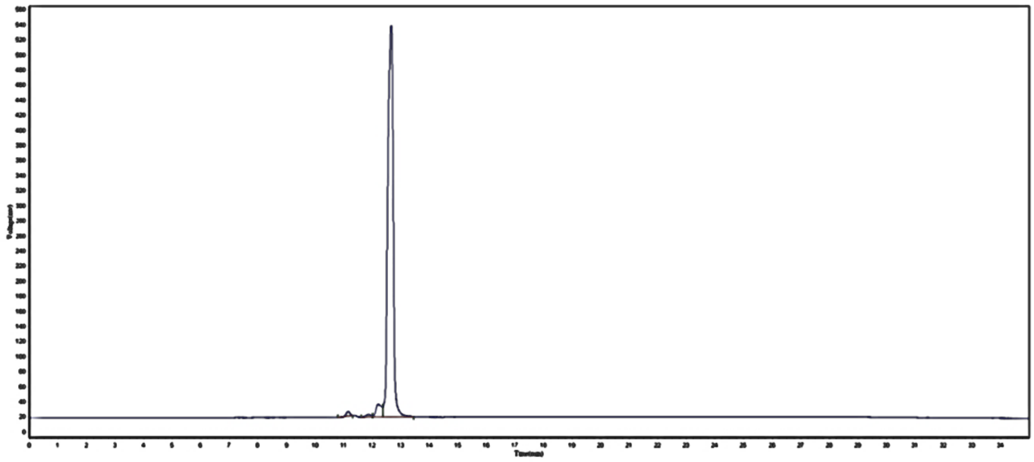

Figure S18. β-5’-d(ACTATTCACCTA) (ODN-**17**)

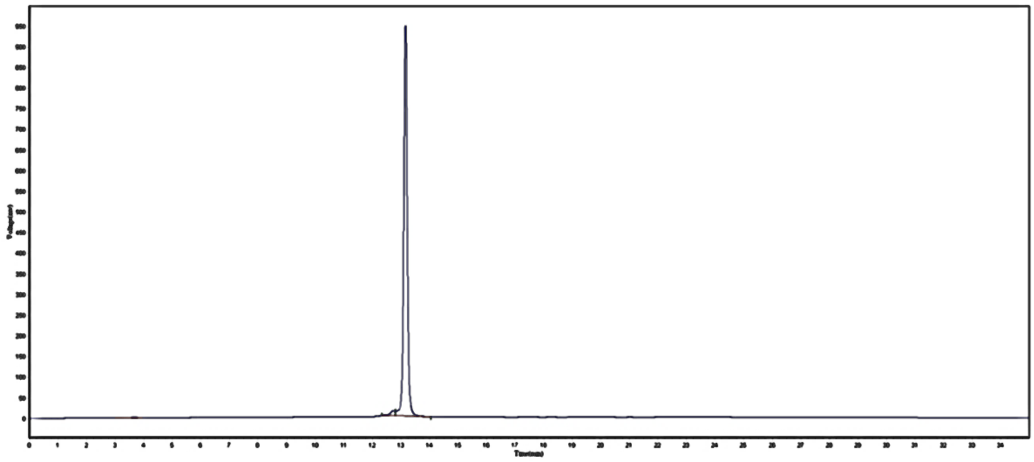

Figure S19. β-5‘-d(A$^{\alpha}$CTATT$^{\alpha}$CACCTA) (ODN-**18**)

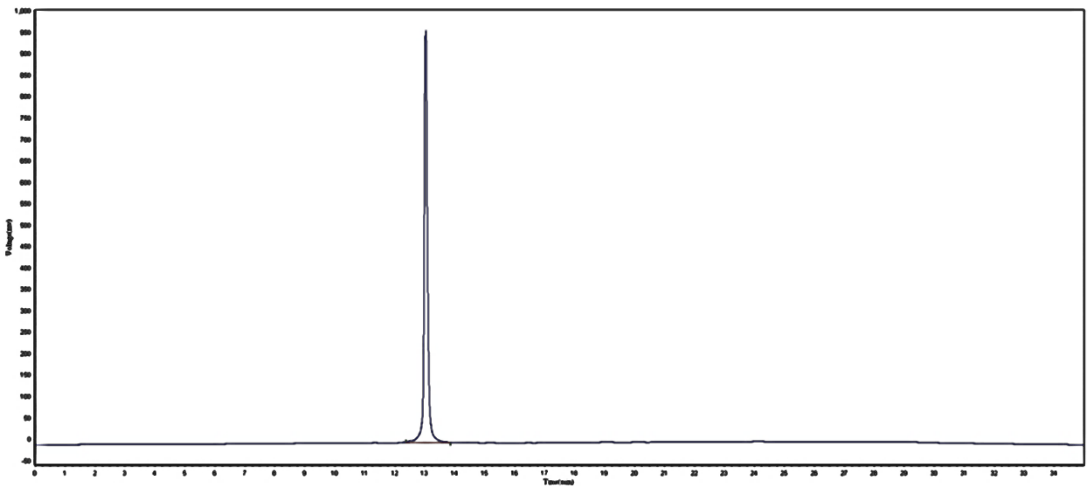

Figure S20. β-5’-d(AGTATT1ACCTA) (ODN-**19**)

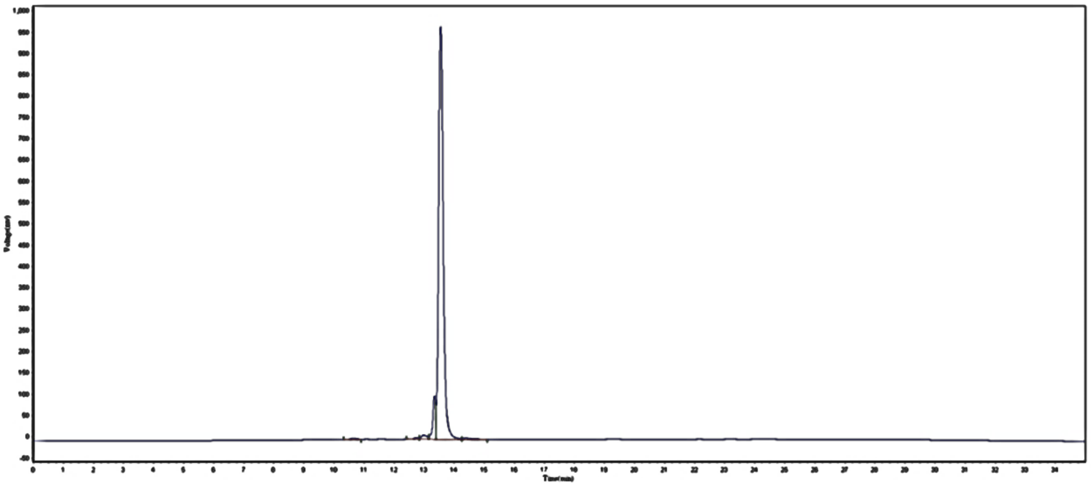

Figure S21. β-5’-d(AGTATT2ACCTA) (ODN-**20**)

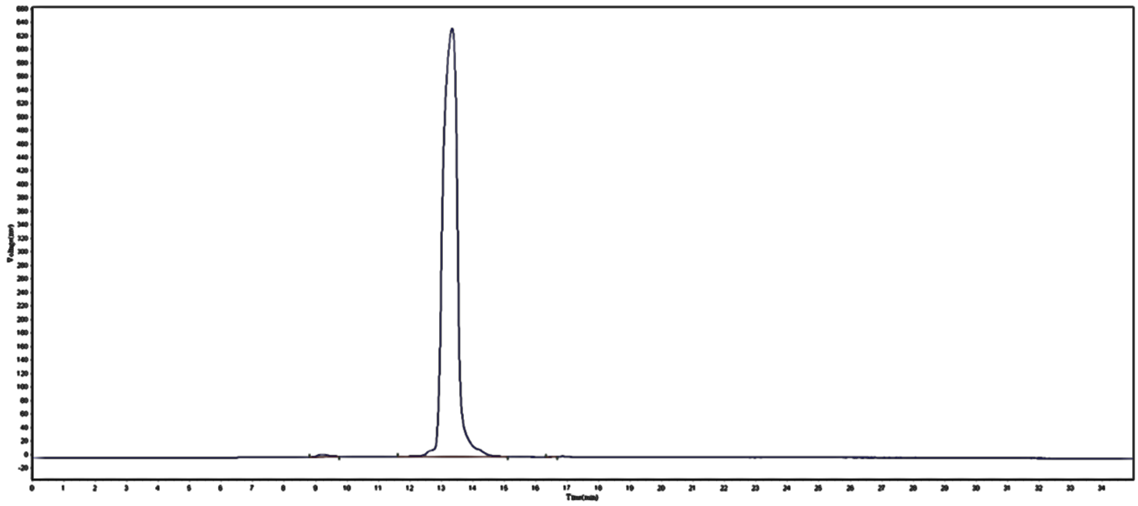

Figure S22. β-5’-d(ATCCACTTATGA) (ODN-**21**)

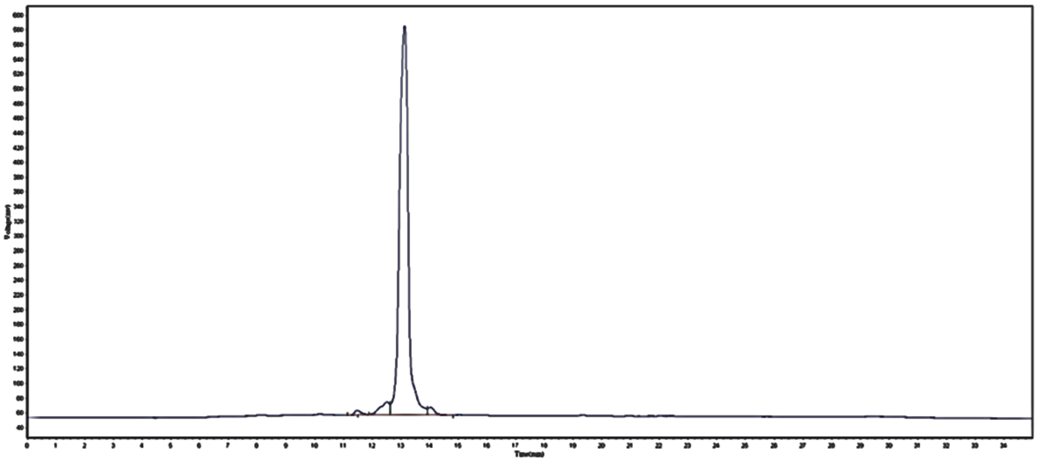

Figure S23. β-5’-d(ATCCA$^{\alpha}$CTTATGA) (ODN-**22**)

Figures S2-S23. Reversed-phase (RP-18) HPLC elution profiles of purified oligonucleotides monitored at 260 nm. X-axis refers to retention time (min); Y-axis refers to UV absorbance at 260 nm, measured in mV. For elution, the following system was used: (A) MeCN, (B) 0.1 M (Et_3NH)OAc (pH 7.0)/MeCN, 95:5; gradient: 0-20 min 0-20% A in B; 20-25 min, 20% A in B; flow rate 0.7 mL/min.

Melting profiles of heterochiral (α/β) oligonucleotide duplexes containing α-dC opposite the canonical bases dG, dA, dC, dT

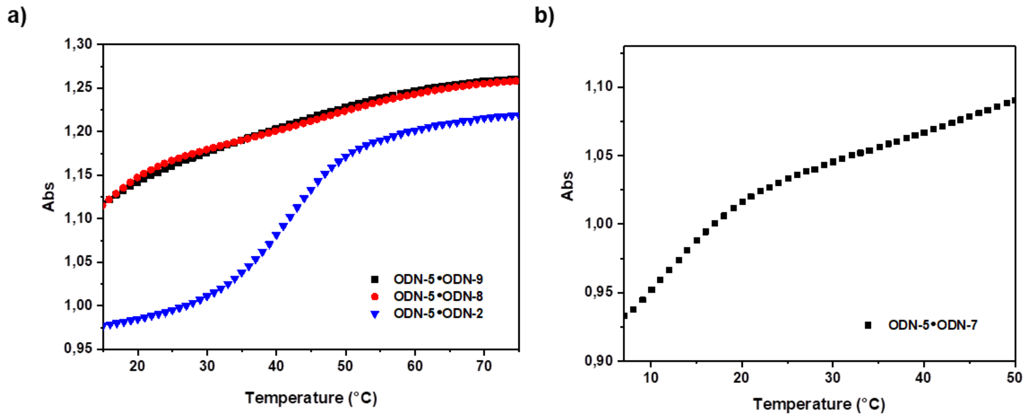

a)

α-5'-d(TCATAACTGGAT) (ODN-**5**)
β-5'-d(AGTATTGACCTA) (ODN-**2**)

α-5'-d(TCATAACTAGAT) (ODN-**5**)
β-5'-d(AGTATTTACCTA) (ODN-**9**)

α-5'-d(TCATAACTGGAT) (ODN-**5**)
β-5'-d(AGTATTAACCTA) (ODN-**8**)

b)

α-5'-d(TCATAACTAGAT) (ODN-**5**)
β-5'-d(AGTATTCATCTA) (ODN-**7**)

Figure S24. Thermal denaturation curves of duplexes a) ODN-**5**•ODN-**2**; ODN-**5**•ODN-**8**; ODN-**5**•ODN-**9**; b) ODN-**5**•ODN-**7** measured with 5 μM + 5 μM single strand concentration in 100 mM NaOAc, 10 mM $Mg(OAc)_2$ buffer (pH = 7.4) at 260 nm.

Melting profiles of heterochiral (α/β) oligonucleotide duplexes containing α-dC opposite the canonical bases dG, dA, dC, dT in the absence and presence of silver ions

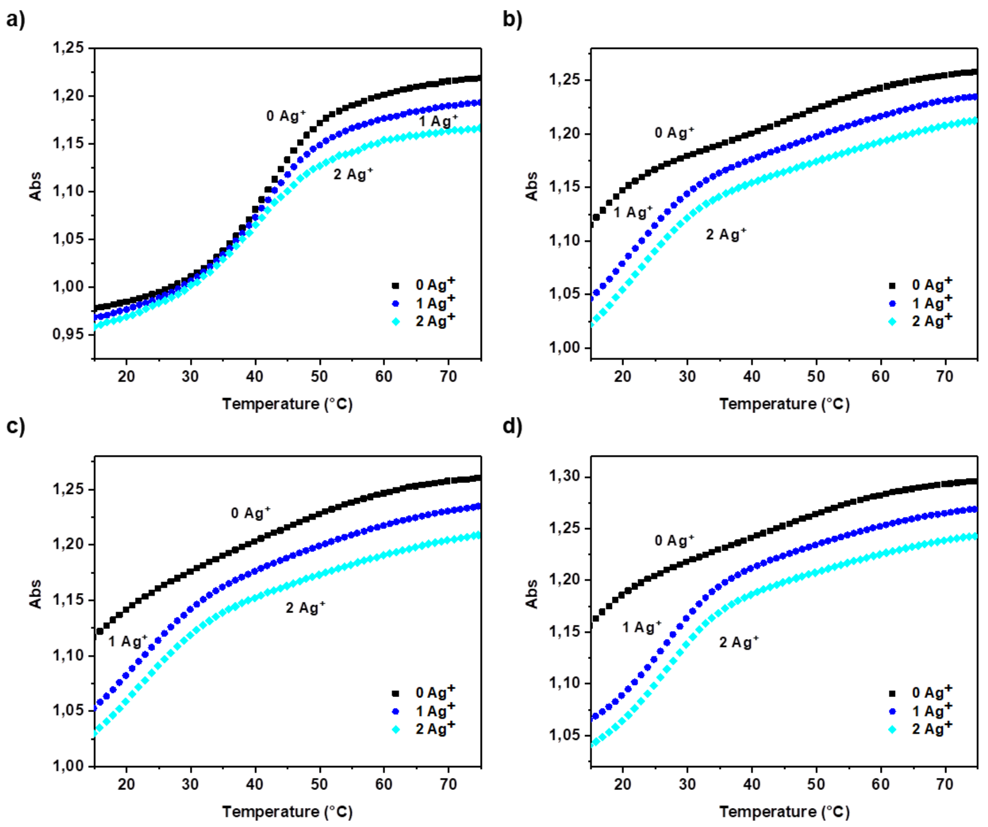

a)	α-5’-d(TCATAACTGGAT) (ODN-**5**)<br>β-5’-d(AGTATTGACCTA) (ODN-**2**)	b)	α-5’d(TCATAACTGGAT) (ODN-**5**)<br>β-5’d(AGTATTAACCTA) (ODN-**8**)
c)	α-5’-d(TCATAACTAGAT) (ODN-**5**)<br>β-5’-d(AGTATTTACCTA) (ODN-**9**)	d)	α-5’-d(TCATAACTAGAT) (ODN-**5**)<br>β-5’-d(AGTATTCATCTA) (ODN-**7**)

Figure S25. Thermal denaturation curves of duplexes a) ODN-**5**•ODN-**2**; b) ODN-**5**•ODN-**8**; c) ODN-**5**•ODN-**9**; d) ODN-**5**•ODN-**7** measured with 5 µM + 5 µM single strand concentration in 100 mM NaOAc, 10 mM $Mg(OAc)_2$ buffer (pH = 7.4) at 260 nm in the presence of various concentrations of Ag^+ (0, 1 and 2 silver ions/duplex).

Melting profiles of heterochiral (α/β) oligonucleotide duplexes containing one or two dC/α-dC or α-dC/α-dC base pairs in the presence and absence of silver ions

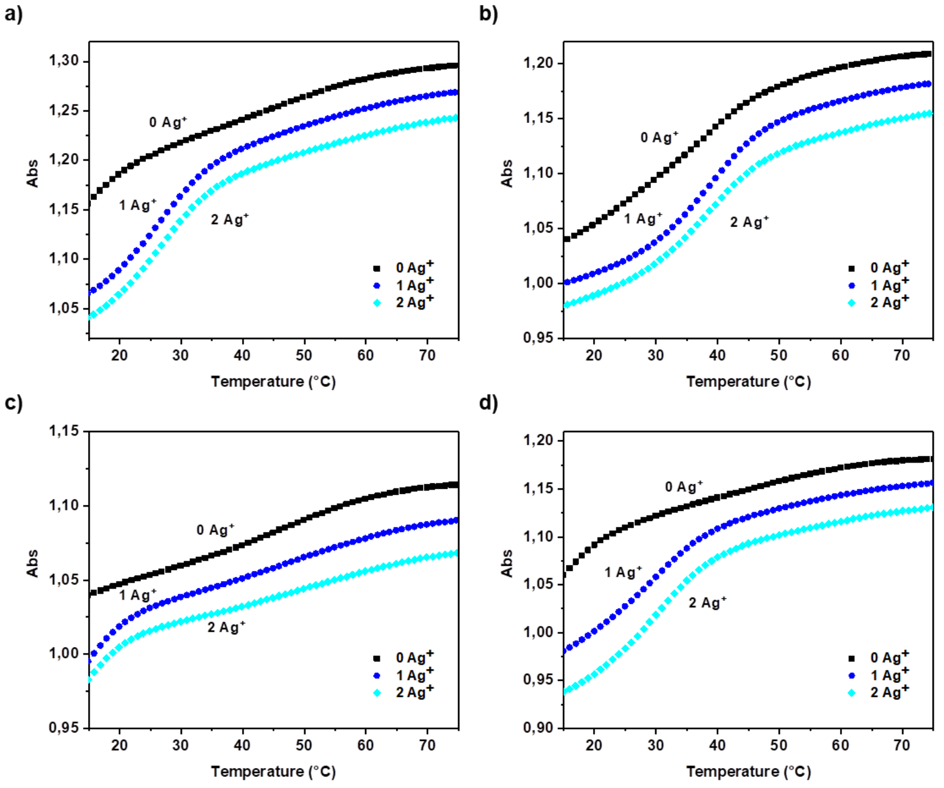

a) α-5'-d(TCATAACTGGAT) (ODN-**5**)
β-5'-d(AGTATTCATCTA) (ODN-**7**)

b) α-5'-d(TCATAACTGGAT) (ODN-**5**)
β-5'-d(AGTATT$^{\alpha}$CACCTA) (ODN-**16**)

c) α-5'-d(TCATAACTAGAT) (ODN-**5**)
β-5'-d(ACTATTCACCTA) (ODN-**17**)

d) α-5'-d(T CATAACTGGAT) (ODN-**5**)
β-5'-d(A$^{\alpha}$CTATT$^{\alpha}$CACCTA) (ODN-**18**)

Figure S26. Thermal denaturation curves of duplexes a) ODN-**5**•ODN-**7**; b) ODN-**5**•ODN-**16**; c) ODN-**5**•ODN-**17**; d) ODN-**5**•ODN-**18** with 5 µM + 5 µM single strand concentration measured in 100 mM NaOAc, 10 mM $Mg(OAc)_2$ buffer (pH = 7.4) at 260 nm in the presence of various concentrations of Ag^+ (0, 1 and 2 silver ions/duplex).

Melting profiles of homochiral (β/β) and heterochiral (α/β) oligonucleotide duplexes containing α-and β-5-iodo substituted dC in the presence and absence of silver ions

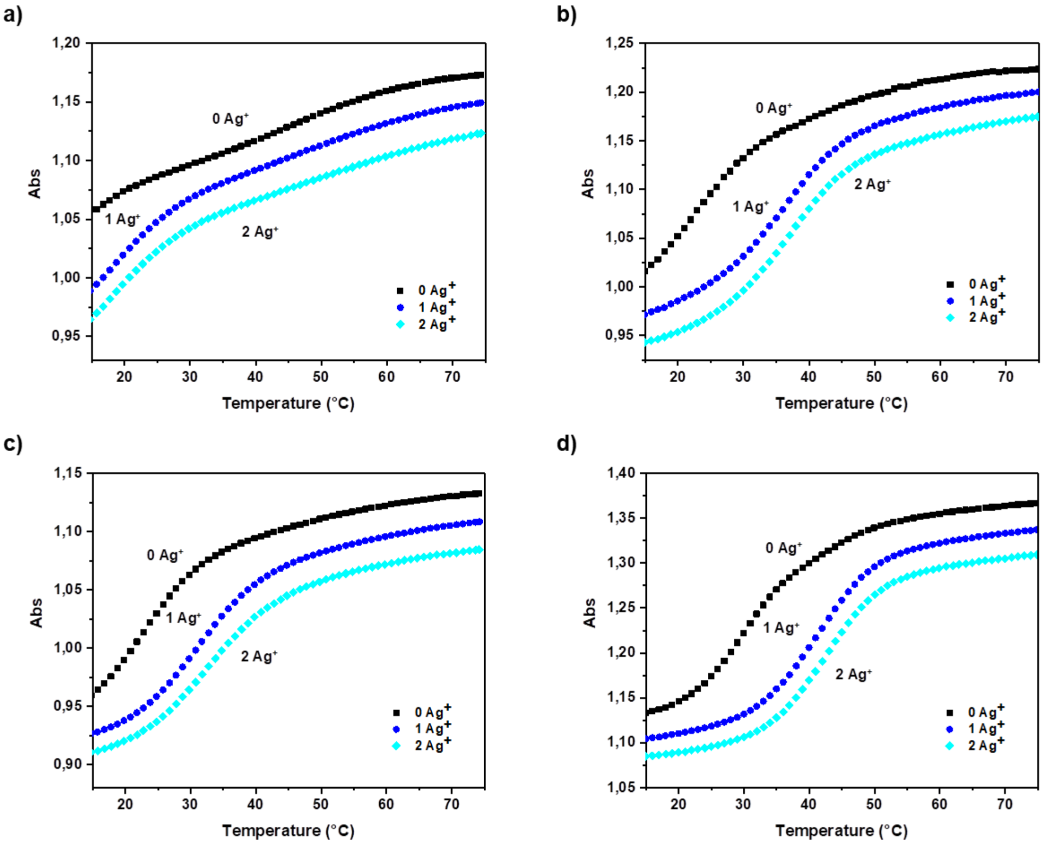

a) α-5’-d(TCATAACTGGAT) (ODN-**5**)
β-5’-d(AGTATT1ACCTA) (ODN-**19**)

b) α-5’-d(TCATAACTGGAT) (ODN-**5**)
β-5’-d(AGTATT2ACCTA) (ODN-**20**)

c) β-5’-d(TAGGTCAATACT) (ODN-**1**)
β-3’-d(ATCCA1TTATGA) (ODN-**19**)

d) β-5’-d(TAG GTCAATACT) (ODN-**1**)
β-3’-d-(ATC CA2TTATGA) (ODN-**20**)

Figure S27. Thermal denaturation curves of duplexes a) ODN-**5**•ODN-**19**; b) ODN-**5**•ODN-**20**; c) ODN-**1**•ODN-**19**; d) ODN-**1**•ODN-**20** with 5 µM + 5 µM single strand concentration measured in 100 mM NaOAc, 10 mM $Mg(OAc)_2$ buffer (pH = 7.4) at 260 nm in the presence of various concentrations of Ag^+ (0, 1 and 2 silver ions/duplex).

Melting profiles of homochiral (β/β) and heterochiral (α/β) oligonucleotide duplexes containing 7-deaza-dA and 7-deaza-dG in the presence and absence of silver ions

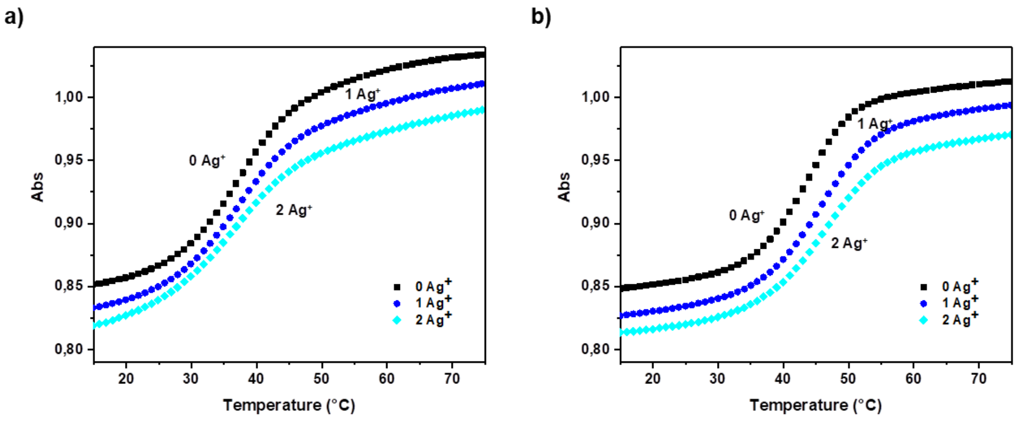

a) α-5'-d(TCATAACTGGAT) (ODN-**5**)
β-5'-d(^{7}A^{7}GT^{7}ATT^{7}G^{7}ACCT^{7}A) (ODN-**10**)

b) β-5'-d(TAGGTCAATACT) (ODN-**1**)
β-3'-d(^{7}ATCC^{7}A^{7}GTT^{7}AT^{7}G^{7}A) (ODN-**10**)

Figure S28. Thermal denaturation curves of duplexes a) ODN-**5**•ODN-**10**; b) ODN-**1**•ODN-**10** with 5 µM + 5 µM single strand concentration measured in 100 mM NaOAc, 10 mM $Mg(OAc)_2$ buffer (pH = 7.4) at 260 nm in the presence of various concentrations of Ag^+ (0, 1 and 2 silver ions/duplex).

Melting profiles of reversed heterochiral (α/β) duplexes containing dC/α-dC or α-dC/α-dC base pairs in the presence and absence of silver ions

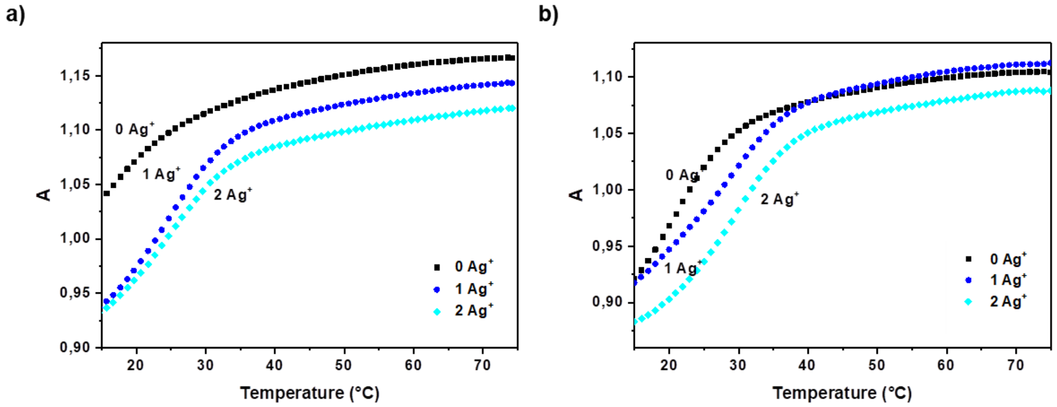

a) α-5'-d(TAGGTCAATACT) (ODN-**6**)
β-5'-d(ATCCACTTATGA) (ODN-**21**)

b) α-5'-d(TAGGTCAATACT) (ODN-**6**)
β-5'-d(ATCCA$^{\alpha}$CTTATGA) (ODN-**22**)

Figure S29. Thermal denaturation curves of duplexes a) ODN-**6**•ODN-**21**; b) ODN-**6**•ODN-**22** with 5 µM + 5 µM single strand concentration measured in 100 mM NaOAc, 10 mM $Mg(OAc)_2$ buffer (pH = 7.4) at 260 nm in the presence of various concentrations of Ag^+ (0, 1 and 2 silver ions/duplex).

Melting profiles and CD spectra of homochiral (β/β) and heterochiral (α/β) oligonucleotide duplexes in the presence and absence of silver ions (0-24 silver ions per duplex)

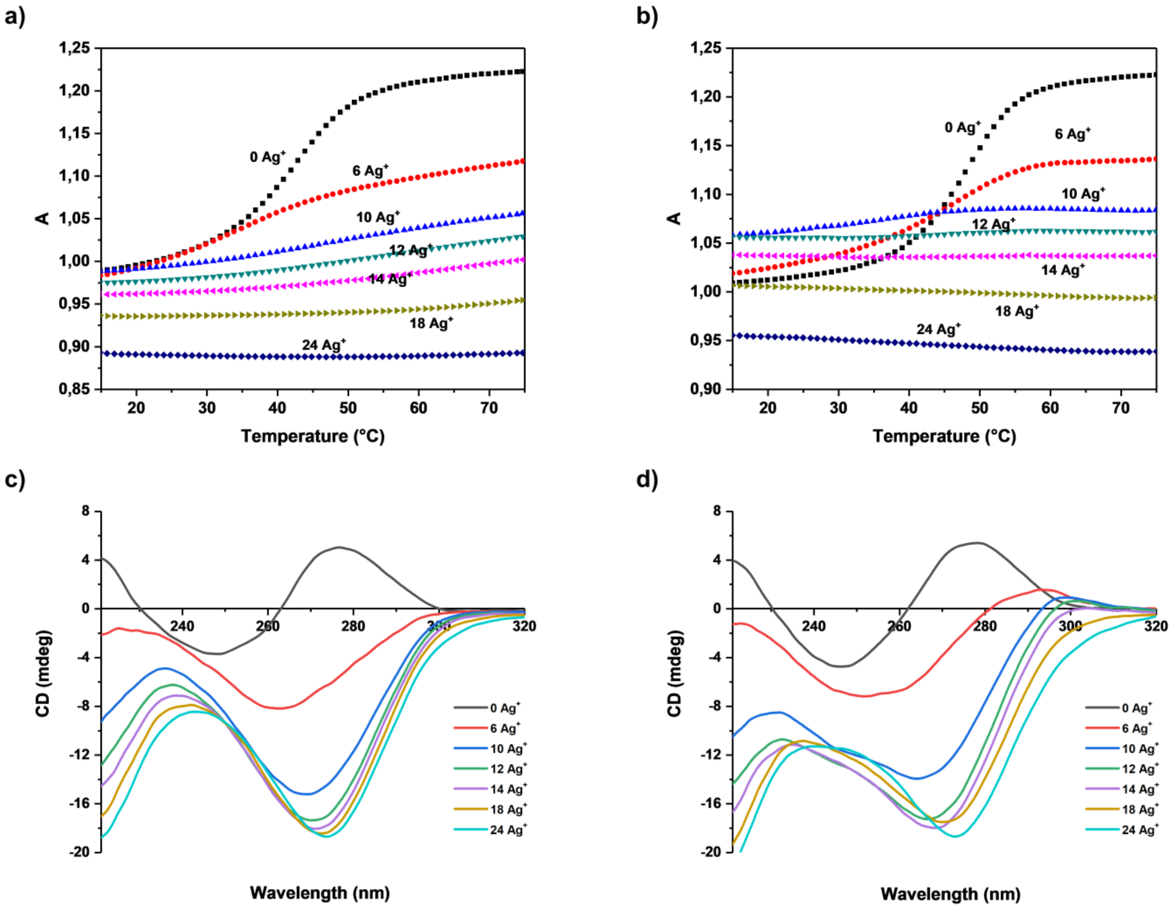

Figure S30. Thermal denaturation curves of duplexes a) ODN-**5**•ODN-**2** and b) ODN-**1**•ODN-**2** with 5 µM + 5 µM single strand concentration measured in 100 mM NaOAc, 10 mM $Mg(OAc)_2$ buffer (pH = 7.4) at 260 nm in the presence of various concentrations of Ag^+ (0-24 silver ions/duplex). CD spectra of duplexes: c) ODN-**5**•ODN-**2** and d) ODN-**1**•ODN-**2**. Measured with 5 µM + 5 µM single strand concentration in 100 mM NaOAc, 10 mM $Mg(OAc)_2$ buffer (pH = 7.4) monitored at 260 nm in the presence of various concentrations of Ag^+ (0-24 silver ions/duplex). The cell path length of the cuvette for the CD spectra was 5 mm.
